# Supplementary material for: Comparative epigenetic analysis of tumour initiating cells and syngeneic EPSC-derived neural stem cells in glioblastoma
Source: Nat Commun. 2021 Oct 21;12:6130. doi: 10.1038/s41467-021-26297-6 (PMC8531305; doi:10.1038/s41467-021-26297-6)
Supplement: Supplementary file 1 — Supplementary Information [file 41467_2021_26297_MOESM1_ESM.pdf]

Supplementary Information

Comparative epigenetic analysis of tumour initiating cells and syngeneic EPSC-derived neural stem cells (SYNGN) in glioblastoma.

Claire Vinel, Gabriel Rosser, Loredana Guglielmi, Myrianni Constantinou, Nicola Pomella, Xinyu Zhang, James R. Boot, Tania A. Jones, Thomas O. Millner, Anaelle A. Dumas, Vardhman Rakyan, Jeremy Rees, Jamie L. Thompson, Juho Vuononvirta, Suchita Nadkarni, Tedani El Assan, Natasha Aley, Yung-Yao Lin, Pentao Liu, Sven Nelander, Denise Sheer, Catherine L.R. Merry, Federica Marelli-Berg, Sebastian Brandner and Silvia Marino

List of supplementary Tables

Table S1: Clinical and molecular data from the 10 patients of the cohort with IDH-wildtype GBM.

Table S2: 11 genes appearing in both datasets DE and DMR and none of those are shared across all patient-derived cell lines

Table S3 Reagents

List of supplementary Data

Supplementary Data 1: Integrated transcriptome and methylome analysis of syngeneic hGIC/iNSC pairs and identification of patient-specific druggable targets (provided separately as an xls file).

List of Supplementary Figures

Fig. S1: Comparative analysis of FFPE bulk tumour and matched-GIC culture and validation of tumor-initiating capacity of GIC in vivo.

Fig. S2: Characterisation of EPSC and EPSC-derived NSC at methylome, transcriptome and molecular level.

Fig. S3: Generation and characterization of EPSC-derived iNSC in mice.

Fig. S4: Transcriptomic analysis of EPSC-derived iNSC and endogenous syngeneic and non-syngeneic brain and non-brain cells.

Fig. S5: Comparative transcriptome analysis and signaling pathways deregulation between GIC, their matched-iNSC and non-syngeneic NSC.

Fig. S6: Integration of inferred cell type proportion of the tumour bulk and pathway enrichment in hGIC vs iNSC.

Fig. S7: GAG analysis and modulation of immune cells migration by GIC as compared to iNSC.

Fig. S8: Characterisation of patient-specific drug sensitivity.

Fig. S9: PTGER4 in GBM and GIC and characterization of PGE1-OH treatment in 2D cultures.

Fig. S10: Epigenetic editing of PTGER4 and CO characterization.

Fig. S11: SYNGLICO characterization and analysis of the effect of PGE1-OH treatment on viability and COGFP+ MFI.

Fig. S12: ALDH3B1/DSF treatment characterisation in 2D culture.

Fig. S13: ALDH3B1/DSF treatment characterisation in syngeneic GLICO model.

Fig. S14: NTRK2/CTX characterisation in 2D culture

Fig.S15 NTRK2/CTX treatment characterisation in SYNGLICO

Fig. S16 Gastro-intestinal drug toxicity assay in patient-specific EPSC derived intestinal organoids.

Table S1: Clinical and molecular data from the 10 patients of the cohort with IDH-wildtype GBM.

GBM No.	Histo ID	Sex	Date of surgery	Age at surgery	Location of tumour	Histology Diagnosis	*IDH1* (R132H IHC)	*ATRX*	*PTEN*	*EGFR*	*IDH1*	*IDH2*	Histone mut (H3F3)
GBM 17	NH15-1661	M	8/19/2015	35	Left frontal	Glioblastoma, *IDH* wild-type	-	Retained	LOH	Strong	-	-	-
GBM 18	NH15-1877	M	9/17/2015	71	Frontal	Glioblastoma, *IDH* wild-type	-	Retained	-	Low	-	-	-
GBM 19	NH15-2101	F	10/12/2015	65	Right parietofrontal	Glioblastoma, *IDH* wild-type	-	Retained	-	-	-	-	-
GBM 26	NH16-270	M	2/2/2016	64	Right frontal	Glioblastoma, *IDH* wild-type	-	Retained	-	Strong	-	-	-
GBM 30	NH16-616	M	3/17/2016	51	Right frontal	Glioblastoma, *IDH* wild-type	-	Retained	-	Strong	-	-	-
GBM 31	NH16-677	F	3/22/2016	70	Right temporal	Glioblastoma, *IDH* wild-type	-	Retained	-	-	-	-	-

GBM 50	NH16 -2063	F	9/6/201 6	49	Right frontal	Glioblast oma, *IDH* wild-type	-	Retained	-	Strong	/	-	-
GBM 52	NH16 -2214	F	9/27/20 16	73	Right frontal	Glioblast oma, *IDH* wild-type	-	Retained	LOH	Strong	-	-	-
GBM 54	NH16 -2255	M	9/30/20 16	66	Right parietal	Glioblast oma, *IDH* wild-type	-	Retained	LOH	Moderate		-	-
GBM 61	NH16 -2806	F	12/1/20 16	66	Right frontal	Glioblast oma, *IDH* wild-type	-	Retained	LOH	Moderate		-	-

-	Negative
/	Not tested

Table S2: 11 genes appearing in both datasets DE and DMR and none of those are shared across all patient-derived cell lines analysed

	DE					DMR				
	**Classification**	**FDR ESC line 1**	**FDR ESC line 2**	**logFC ESC line 1**	**logFC ESC line 2**	**Classification**	**FDR ESC line 1**	**FDR ESC line 2**	**Median delta M ESC line 1**	**Median delta M ESC line 2**
**Patient 19**										
CIDEB	upregulated	0.00861	0.0025	10.5746	10.5746	hypomethylated	7E-05	1.9E-09	-2.29479	-2.87426
SDR42E1	upregulated	9.6E-06	5.2E-08	7.89926	6.7734	hypomethylated	0.00538	4.8E-06	-2.9875	-3.16119
**Patient 50**										
HOXB4	upregulated	0.00692	0.00871	6.7055	4.59744	hypomethylated	0.00384	0.00032	-2.09186	-1.53369
**Patient 54**										
ANKRD6	upregulated	0.00719	0.00316	2.19287	2.21611	hypomethylated	0.00661	0.00361	-3.39798	-2.91449
BMPR1B	Upregulated	0.00042	0.00036	3.99535	3.39781	hypomethylated	0.00363	1.3E-05	-3.94449	-4.34084
CIDEB	Upregulated	0.00879	0.00414	9.09057	9.09057	hypomethylated	0.00075	2.5E-08	-2.84122	-3.11284
DCDC2	Upregulated	6.5E-07	4.6E-06	5.38218	3.74065	hypomethylated	0.00363	5.2E-06	-3.94753	-3.39403
HOXA3	Upregulated	0.00175	0.00119	10.8768	5.88029	hypomethylated	0.00055	1.6E-05	-2.62662	-1.92062
LRRTM2	Upregulated	3.6E-05	1.2E-05	6.23953	4.76291	hypomethylated	0.00324	1.9E-08	-2.8131	-3.34121
NR2F2	Upregulated	0.00019	0.00022	10.1096	5.73603	hypomethylated	0.00549	0.00323	-2.50875	-1.74077
NR2F2-AS1	Upregulated	0.00207	0.00364	9.91577	5.10696	hypomethylated	0.00549	0.00323	-2.50875	-1.74077
PALMD	Upregulated	1.3E-05	1.8E-06	7.82612	5.62443	hypomethylated	0.00363	0.00066	-3.33318	-2.74424

Experimental procedures

**PRIMERS**	**SEQUENCE** Forward (5'-3')	**SEQUENCE** Reverse (5'-3')
h/mACTB	GCGAGAAGATGACCCAGATC	CCAGTGGTACGGCCAGAGG
hSOX2endo	ACAGCAAATGACAGCTGCAAA	AAGTCCAGGATCTCTCTCATAAAAGTTT
hPOU5F1endo	CACTGTACTCCTCGGTCCCTTTC	CAACCAGTTGCCCCAAACTC
h/mATP5B	CCCAGGCTGGTTCAGAGGT	AGGGGCAGGGTCAGTCAAG
hNANOG	AGAAAAACAACTGGCCGAAGAAT	GTTGAATTGTTCCAGGTCTGGTT
h/mPAX6	AACGATAACATACCAAGCGTGT	GGTCTGCCCGTTCAACATC
h/mGATA6	GCCAACTGTCACACCACAAC	TCATAGCAAGTGGTCTGGGC
h/mGSC	ACAACAACTACTTCTACGGGC	CCCACGTTCATGTAGGGCA
mGATA6	CAGGTCAAGACGGCCTCTAC	AAGAATCCTGTCGCACGGAG
mNANOG	TTGCTTACAAGGGTCTGCTACT	ACTGGTAGAAGAATCAGGGCT
mPOU5F1	TAGGTGAGCCGTCTTTCCAC	GCTTAGCCAGGTTCGAGGAT
*mSOX2-endo_*	TTAACGCAAAAACCGTGATG	GAAGCGCCTAACGTACCACT
hPTGER4	CCGGCGGTGATGTTCATCTT	CCCACATACCAGCGTGTAGAA
hNTRK2	ATCTCCAACCTCAGACCACCACT	AATCTGTTTCTCATCCTTCCCATAC
h/mGAPDH	CTGAGGCTCCCACCTTTCTC	TTATGGGAAAGCCAGTCCCC
hALDH3B1	ACAAGTCAGCCTTCGAGTCGG	AGCACCACACAGTTCCCTGC
h/ALDH1A2	AAGCTGGGACTGTTTGGATCA	TACTCCCGCAAGCCAAATTC
h/ALDH1A3	ACGGTCTGGATCAACTGCTA	CCGTCCGATGTTTGAGGAAG
h/ALDH1B1	AGACGGTCACCATCAAGGTT	AGCATTCGTCAAGGTGGTTG
h/ALDH1L2	GCTTTCCAAAGGGGGTCATC	GCTAACAGCACAGCTCTTCAT
h/ALDH2	GGGAGAGCCAACAATTCCAC	CCACTCCCCGACATCTTGTA
h/ALDH3A1	ATCGCCTGGGGGAAATTCAT	AGTCCCGGGATTTCTTAGCA
h/ALDH3A2	TTGGTACTTCCCAGGGCTAC	GGTCAAGTCCTTGAGTCCCA
h/ALDH4A1	AGCCTCTGGAACCAATGACA	CACCTGGACGGACAGACAG
h/ALDH5A1	GACGAAGCACCTTCCTTTCC	ATAGCTTCCCAGTGGCTCAA
h/ALDH6A1	TCACCGCTTTTGGTTGATCC	TGTGGGATAAAAGAGGGGCT
h/ALDH7A1	GGTTGCCCTTGGATCTGTTC	TGAACTTTGCCCAGCTCTCT
h/ALDH9A1	AGACGACATGACCTGTGTGA	CCGTTGGATGTCCCTGGTAA
h/ALDH16A1	TTCGGATCAGCCCAGGGTTC	TCAGGCATCAGTCCCCCATA
h/ALDH18A1	CCTGCAGGGGGTAAATGTTATT	TCACAGACTGCTGATCTCCG
**PLASMIDS**	**ORIGIN**	**FLUORESCENT REPORTER/SELECTION**
pCMV-VSVG	Addgene, 8454	NA
pCMV-HIV1	Addgene	NA
Ex-NEG-Lv122	GeneCooeia	GFP/Puromycin
EX-Q0086-Lv122	GeneCooeia	GFP/Puromycin
shNTRK2	Horizon-Dharmacon	GFP/Puromycin
pLoxGFP	Addgene, 12241	FDP
FUW-dCas9-Tet1CD-HA-P2A-tagBFP	Addgene, 108246	BFP
pgRNA-humanized	Addgene, 44248	mCherry/Puromycin

**LENTIVIRUS**		
shALDH3B1	Horizon-Dharmacon	GFP/Puromycin
**PLASMID GUIDES**	Forward primer (5’-3’)	Reverse primer (5’-3’)
gRNA 1 (g1): TAAAAGGAGTTGGAGTCAGT	TTGGTAAAAGGAGTTGGAGTCAGT	AAACACTGACTCCAACTCCTTTTA
gRNA 2 (g2): CATCTAGATCGTGGAAGAGA	TTGGCATCTAGATCGTGGAAGAGA	AAACTCTCTTCCACGATCTAGATG
gRNA 3 (g3): AAGAAATAGTACACTCCGAG	AAGAAATAGTACACTCCGAG	TTGGAAGAAATAGTACACTCCGAG
gRNA 4 (g4): TTCCCACTCCGCACCTCCGA	TTGGTTCCCACTCCGCACCTCCGA	AAACTCGGAGGTGCGGAGTGGGAA
gRNA 5 (g5): CCATTGGCCGGATTGGAAGG	TTGGCCATTGGCCGGATTGGAAGG	AAACCCTTCCAATCCGGCCAATGG
gRNA 6 (g6): AGAAAAGTTTGTACAGAGGG	TTGGAGAAAAGTTTGTACAGAGGG	AAACCCCTCTGTACAAACTTTTCT
**PRIMARY ANTIBODIES**	**Reference**	
Vimentin	Abcam ab137321	
NANOG	Biolegend #16H3A38	
SOX2	sc-17320	
Oct3/4	sc-8628	
SSEA4 AF647	BD-560796	
TRA 1-60	sc-21705	
alpha-fetoprotein	R&D-MAB1368	
Beta 3 tubulin	R&D-MAB1195	
alpha-smooth muscle	R&D-MAB1420	
hNestin	R&D MAB5326	
mNestin	Abcam ab6142	
NTRK2	Abcam ab33655	
PTGER4	LS-B6947-1	
Chondroitin sulfate (CS-56)	Abcam ab11570	
CD8 AF700	Biolegend-301028	
CD4 PE	Biolegend-317409	
CD25 PE-Cy7	Biolegend-356108	
CD127 APC	Biolegend-351316	
Foxp3 eF450	Thermo Fisher-48-4776-42	
CD56 FITC	Biolegend-304603	
CD14 Percp Cy5,5	eBioscience-45-0149-41	
TUJ1	Abcam Ab7751	
GFAP	Dako 20025480	
Ki67	Abcam ab15580	
Cleaved-Caspase3	Cell Signaling 9664	
Villin	Santa Cruz sc58897	

E-Cadherin	BD 610181	
CDX2	R&D AF3665	
NeuN	ab104225	
GFP	Millipore AB16901	
**COMPOUNDS**	Reference	
Chondroitinase ABC	Sigma C3667	
PGE1-OH	CAY13020	
Cyclotraxin-B	Tocris-5062	
Disulfiram	Sigma PHR1690	

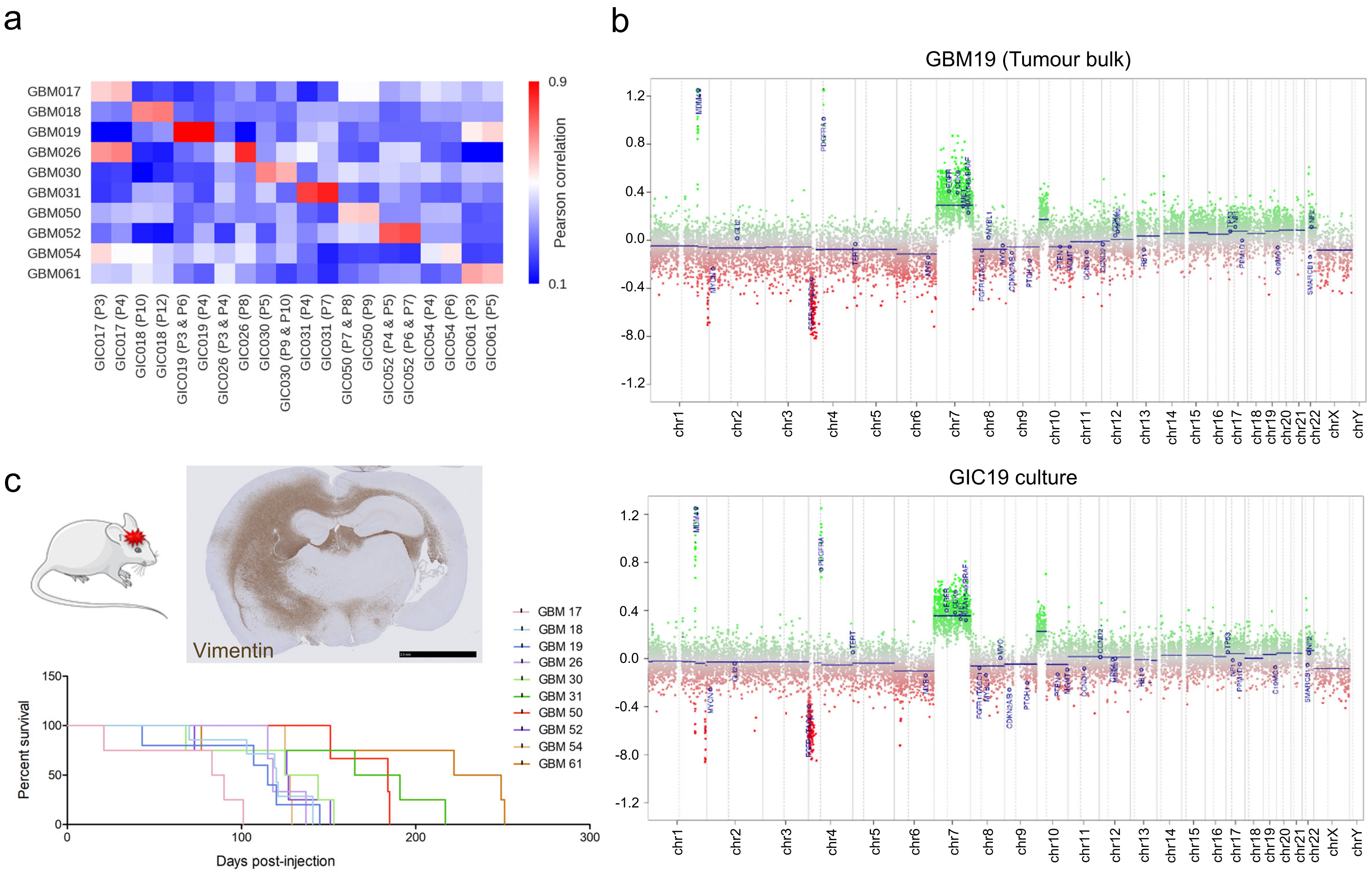
a
GBM017
GBM018
GBM019
GBM026
GBM030
GBM031
GBM050
GBM052
GBM054
GBM061
GIC017 (P3)
GIC017 (P4)
GIC018 (P10)
GIC018 (P12)
GIC019 (P3 & P6)
GIC019 (P4)
GIC026 (P3 & P4)
GIC026 (P8)
GIC030 (P5)
GIC030 (P9 & P10)
GIC031 (P4)
GIC031 (P7)
GIC050 (P7 & P8)
GIC050 (P9)
GIC052 (P4 & P5)
GIC052 (P6 & P7)
GIC054 (P4)
GIC054 (P6)
GIC061 (P3)
GIC061 (P5)
0.9
Pearson correlation
0.1
b
GBM19 (Tumour bulk)
GIC19 culture
1.2
0.8
0.4
0.0
-0.4
-8.0
-1.2
chr1
chr2
chr3
chr4
chr5
chr6
chr7
chr8
chr9
chr10
chr11
chr12
chr13
chr14
chr15
chr16
chr17
chr18
chr19
chr20
chr21
chr22
chrX
chrY
c
Vimentin
Percent survival
150
100
50
0
Days post-injection
0
100
200
300
GBM 17
GBM 18
GBM 19
GBM 26
GBM 30
GBM 31
GBM 50
GBM 52
GBM 54
GBM 61

Fig. S1: Comparative analysis of FFPE bulk tumour and matched-GIC culture and validation of tumor-initiating capacity of GIC in vivo.

a) Pairwise Pearson correlation heatmap based on the gene expression of GBM bulk tissue versus GIC (Gliobastoma-Initiating Cells). The highest correlation in each row corresponds to the matching GIC line in all but one case (patient 17).

b) Genome wide copy number plot shows matching profiles in GBM-bulk and GIC (patient 19), a representative example is shown here. Green shades indicate amplifications while red indicates deletions.

c) Representative image of xenograft brain tumour tissue stained with human vimentin and survival curves of xenograft models generated from each GIC patient derived line. GIC26 and 50: n=3, GIC30, 31, 17, 52 54 and 61: n=4, GIC19: n=5 and GIC18: n=7 mice were injected per line. 100% of the mice developed tumours.

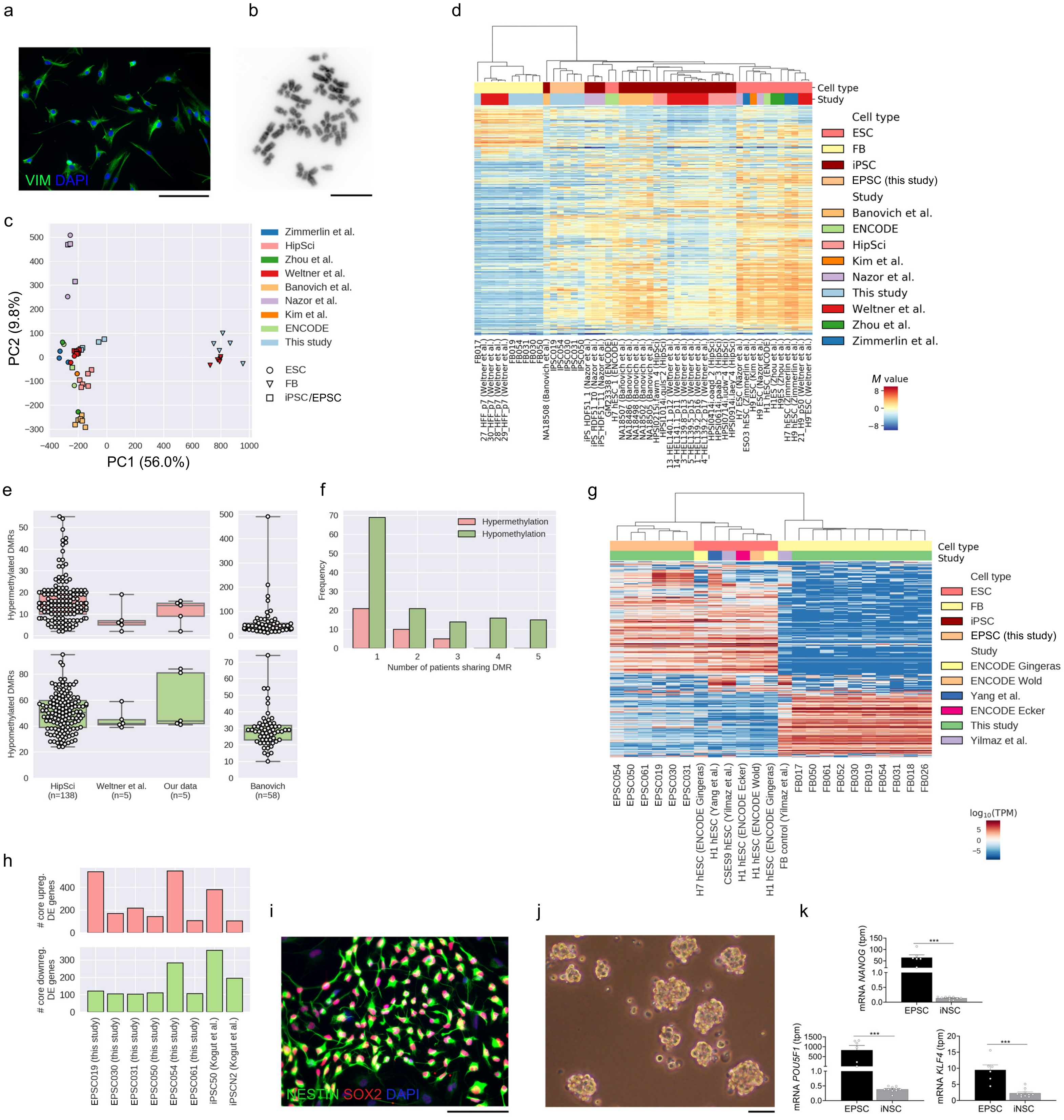

a
VIM DAPI
b
c
Zimmerlin et al.
HipSci
Zhou et al.
Weltner et al.
Banovich et al.
Nazor et al.
Kim et al.
ENCODE
This study
ESC
FB
iPSC/EPSC
PC2 (9.8%)
PC1 (56.0%)
d
Cell type
Study
Cell type
ESC
FB
iPSC
EPSC (this study)
Study
Banovich et al.
ENCODE
HipSci
Kim et al.
Nazor et al.
This study
Weltner et al.
Zhou et al.
Zimmerlin et al.
M value
e
Hypermethylated DMRs
Hypomethylated DMRs
HipSci (n=138)
Weltner et al. (n=5)
Our data (n=5)
Banovich (n=58)
f
Hypermethylation
Hypomethylation
Frequency
Number of patients sharing DMR
g
Cell type
Study
Cell type
ESC
FB
iPSC
EPSC (this study)
Study
ENCODE Gingeras
ENCODE Wold
Yang et al.
ENCODE Ecker
This study
Yilmaz et al.
EPSC054
EPSC050
EPSC061
EPSC019
EPSC030
EPSC031
H7 hESC (ENCODE Gingeras)
H1 hESC (Yang et al.)
CSES9 hESC (Yilmaz et al.)
H1 hESC (ENCODE Ecker)
H1 hESC (ENCODE Wold)
H1 hESC (ENCODE Gingeras)
FB control (Yilmaz et al.)
FB017
FB050
FB061
FB052
FB030
FB019
FB054
FB031
FB018
FB026
log10(TPM)
h
core upreg. DE genes
core downreg. DE genes
EPSC019 (this study)
EPSC030 (this study)
EPSC031 (this study)
EPSC050 (this study)
EPSC054 (this study)
EPSC061 (this study)
iPSC50 (Kogut et al.)
iPSCN2 (Kogut et al.)
i
NESTIN SOX2 DAPI
j
k
mRNA NANOG (tpm)
mRNA POU5F1 (tpm)
mRNA KLF4 (tpm)
EPSC
iNSC
***

Fig. S2: Characterisation of EPSC and EPSC-derived NSC at methylome, transcriptome and molecular level.

a) Human vimentin immunofluorescence (green) staining of fibroblasts isolated from the dura mater. Scale bar is 50 µm. Staining have been performed on the fibroblasts of each of the 10 patients with consistent results.

b) Representative image (iNSC18) of a normal karyotype as assessed on chromosomal spread. Scale bar is 5 µm. Chromosomal spreads have been performed on fibroblasts of each of the 10 patients with consistent results.

c) Principal component analysis plot of the gene expression profiles for our EPSC (Expanded Potential Stem Cells) lines, published reference ESC (Embryonal Stem Cells) and fibroblasts. The first two components are shown.

d) Hierarchical clustering of our EPSC compared to the patient-matched fibroblast of origin and a reference lines on the basis of gene expression from RNA sequencing data.

e) Boxplot showing the number of hyper- and hypomethylated Differentially Methylated Regions (DMRs) identified in different published cohorts of iPSC (induced Pluripotent Stem Cells) lines relative to reference ESC lines. Boxplots show minima and maxima (whiskers), interquartile range (shaded area) and median (horizontal line). Overlaid markers show all data points (some overlapping markers have been removed for visual clarity where n is large). Note that the y axis differs for the Banovich cohort. Each point in the overlaid swarm plot represents a single iPSC line. (HipSci: n=138, Weitner et al: n=5 and our data: n=5 independent lines).

f) Bar chart quantifying the number of hyper- and hypomethylated DMRs identified in our EPSC lines relative to reference ESC separated by the number of patients they present in. Since we consider 5 lines in total, the right most bar refers to DMRs shared between all of them.

g) Hierarchical clustering of the gene expression profiles of our EPSC and fibroblast (FB) lines together with reference ESC datasets on the basis of gene expression from RNA sequencing data.

h) Bar chart quantifying the number of up- and downregulated Differentially Expressed (DE) genes identified in our EPSC lines and reference iPSC lines relative to reference ESC.

i) Co-expression of NESTIN (green) and SOX2 (red) in iNSC (left). Scale bar is 50 µm. Staining have been performed on each of the 10 iNSC lines with consistent results.

j) Formation of neurosphere, representative brightfield image. Scale bar is 50 µm. Experiments have been performed on each of the 10 iNSC lines with consistent results.

k) Expression of pluripotency markers *NANOG*, *POU5F1* and *KLF4* in EPSC (black bars) and iNSC (grey bars). Results are expressed as tpm from the RNA sequencing analysis (n=6 for EPSC and n=10 for iNSC, *t* test, *p* value (*NANOG*)<0,0001, *p* value (*POU5F1*)=0,0003, *p* value (*KLF4*)<0,0001).

All graphs report mean ±SEM. Statistical significance for all panels *$p \leq 0.05$, **$p \leq 0.01$, ***$p \leq 0.001$. Source data are provided in the source data file.

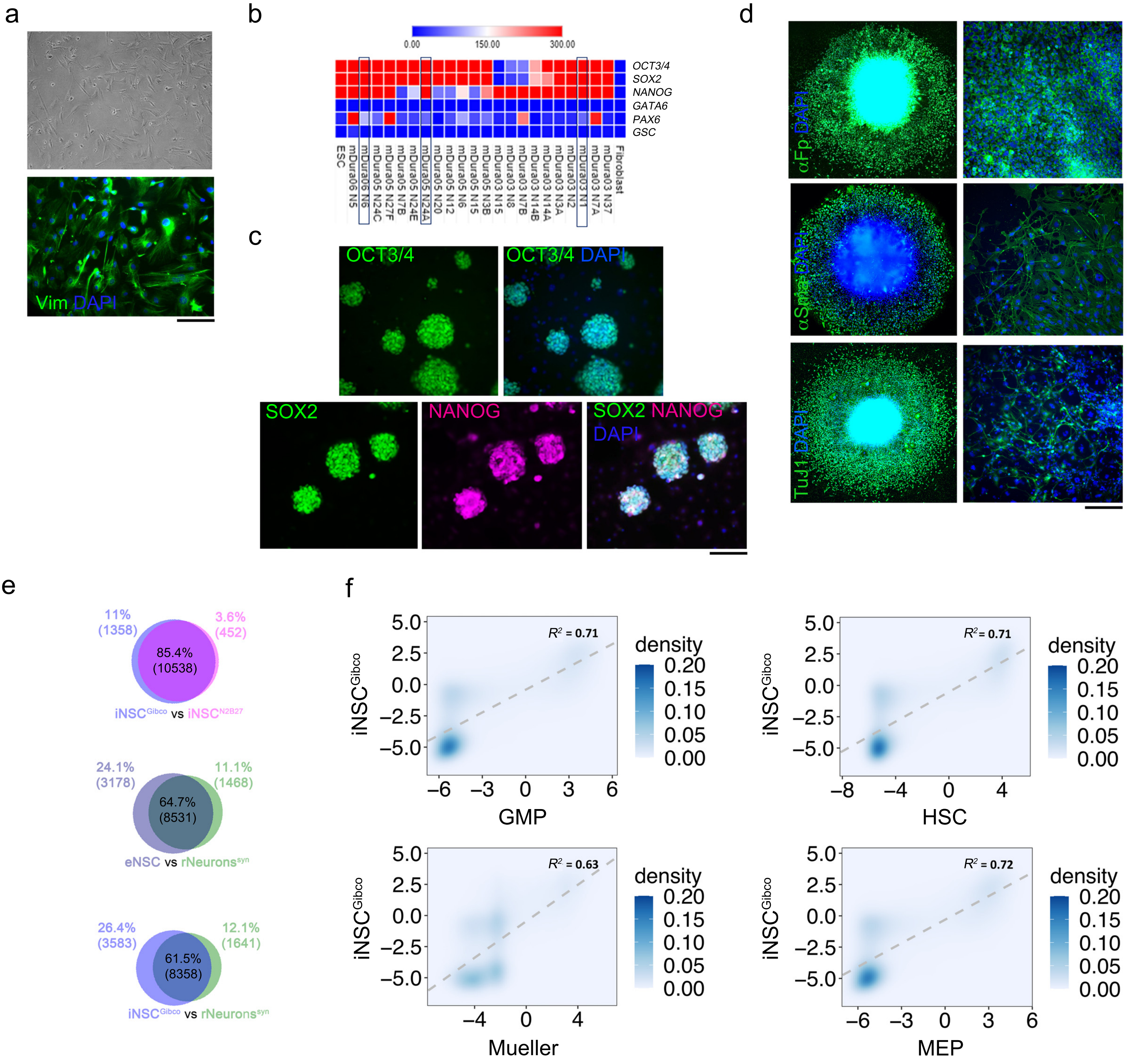

g

	Results	*p* Chi	Effect size
Low iNSC^Gibco	Low eNSC	0.11	0.01
	Low Mueller	4E-166	0.091
	Low Satellite	3.3E-135	0.083
	Low HSC	3.67E-05	0.018
	Low GMP	0.011	0.013
	Low MEP	0.028	0.012
High iNSC^Gibco	High eNSC	0.004	0.025
	High Mueller	2.93E-49	0.105
	High Satellite	5.66E-42	0.096
	High HSC	7.4E-46	0.091
	High GMP	9.78E-39	0.084
	High MEP	1.17E-37	0.083

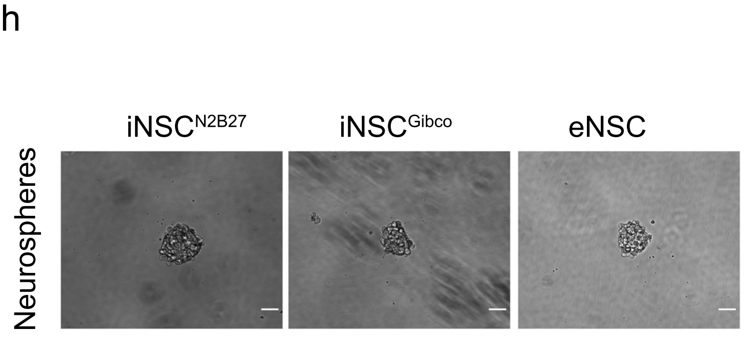

Fig. S3: Generation and characterization of EPSC-derived iNSC in mice.

a) Brightfield (top) and immunostaining for vimentin (green, bottom) of fibroblast cultures isolated from dura mater of 3 months old C57Bl6 mice. Scale bar is 20 µm. Staining have been performed on fibroblasts from the 3 mice with consistent results.

b) Heatmap showing mRNA expression of stemness markers (*OCT3/4*, *SOX2*, *NANOG*) and differentiation markers (*GATA6*, *PAX6, GSC*) for EPSC (Expanded Potential Stem Cells) colonies normalised on the respective fibroblasts (mice 3, 5, 6) and compared to reference ESC (Embryonal Stem Cells). Squares indicate EPSC with high expression of stemness markers and low expression of the differentiation markers, which were induced into iNSC (induced Neural Stem Cells).

c) Expression of OCT3/4 (green, top) and SOX2 (green, bottom), NANOG (purple, bottom) in EPSC colonies. Scale bar is 20 µm. Experiments have been performed on 3 EPSC lines with consistent results.

d) EPSC-derived EB (embryoid bodies) immunostained for markers of the three germ layers: Alpha Fetoprotein (endoderm, top), alpha Smooth Muscle Actin (mesoderm, middle),and β3Tubulin (ectoderm, bottom). Scale bar is 400 µm (left) and 20 µm (right). Experiments have been performed on 3 EPSC lines with consistent results.

e) Venn diagram of overlapping genes (TPM >1) from $iNSC^{Gibco}$ and $iNSC^{N2B27}$, or neurons and from eNSC and neurons; results are expressed in percentages of genes shared or specific to each cell type.

f) Two-dimensional density plots of DNA methylation profiles (*M*-values) between $iNSC^{Gibco}$ and 4 different cell types: granulocyte-macrophage progenitor (GMP) (top left), Mueller cells (bottom left), hematopoietic stem cells (HSC) (top right) and megakaryocyte/erythrocyte progenitors (MEP) (bottom right). A median distribution of *M*-values has been obtained for each cell type across replicas before performing Spearman's correlation. The R^2 value is shown for each plot. (e/i/r) NSC: (endogenous/ induced/reference) Neural Stem Cells; rNeurons: reference Neurons; Gibco: EPSC neural induction with Gibco commercial protocol. N2B27: EPSC neural induction with bespoke published protocol[32]. Syn:syngeneic. Non Syn: non syngeneic.

g) Statistical comparison between the relative abundance profiles of annotated regions, separately for methylated (M>0) and unmethylated (M<0) CpG sites. The reference cell is $iNSC^{Gibco}$. $\chi 2$ test *p*values and effect size quantify how significantly different the two profiles under examination are: low p-values and high effect sizes are indices of strong differences.

h) Neurospheres generated from eNSC (endogenous neural stem cells) and iNSC 3 days post seeding at single cell dilution. Scale bar is 50 µm. Experiments have been performed on iNSC from 3 mice with consistent results.

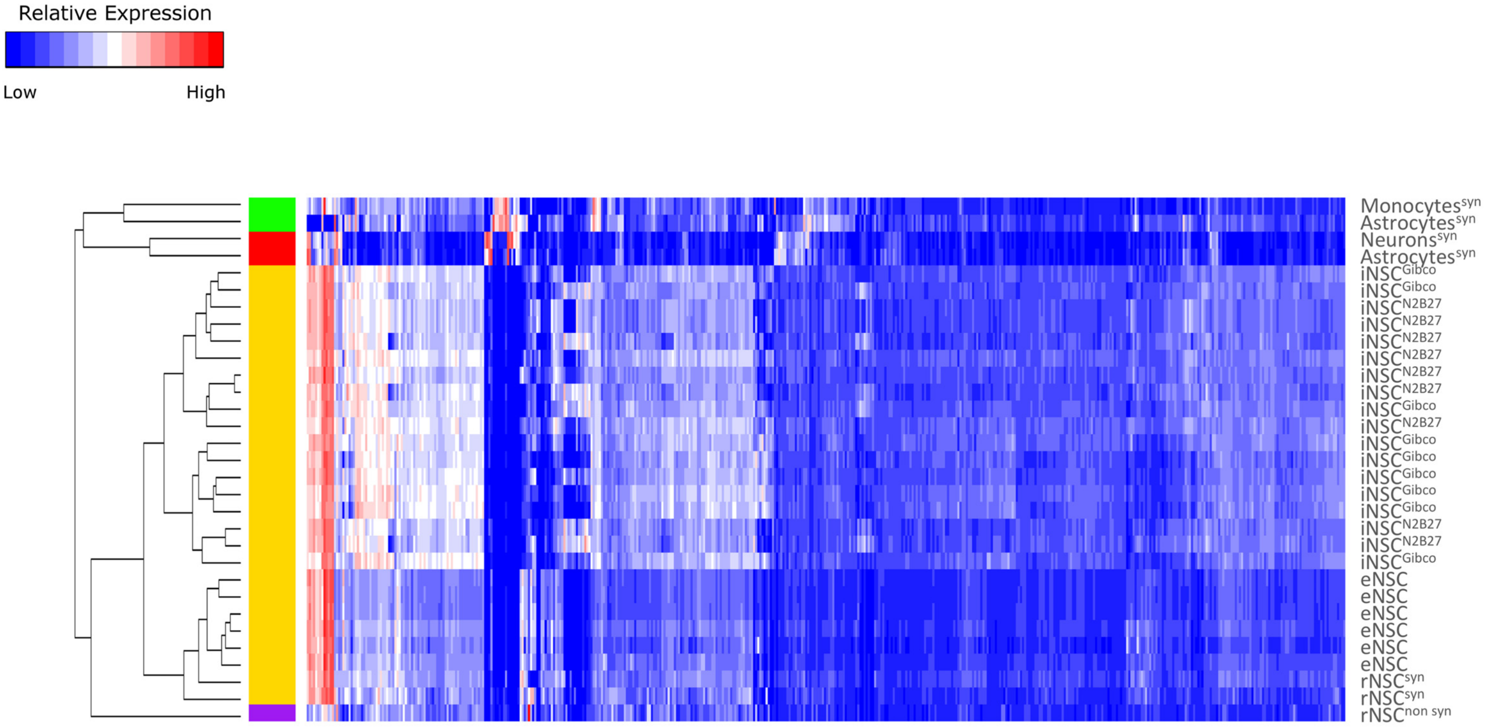

Relative Expression
Low
High
Monocytes^syn
Astrocytes^syn
Neurons^syn
Astrocytes^syn
iNSC^Gibco
iNSC^Gibco
iNSC^N2B27
iNSC^N2B27
iNSC^N2B27
iNSC^N2B27
iNSC^N2B27
iNSC^N2B27
iNSC^Gibco
iNSC^N2B27
iNSC^Gibco
iNSC^Gibco
iNSC^Gibco
iNSC^Gibco
iNSC^Gibco
iNSC^N2B27
iNSC^N2B27
iNSC^Gibco
eNSC
eNSC
eNSC
eNSC
eNSC
eNSC
rNSC^syn
rNSC^syn
rNSC^non syn

Fig. S4: Transcriptomic analysis of EPSC-derived iNSC and endogenous syngeneic and non-syngeneic brain and non-brain cells.

Hierarchical clustering and heatmap representing scaled gene expression from RNA sequencing of eNSC, iNSC^{Gibco}, iNSC^{N2B27} as well as rNSC^{syn} and rNSC$^{non\ syn}$, astrocytes, monocytes and neurons (top 500 genes). The dendrogram has been produced separately, with the normalized gene expression of the full list of genes. The vertical colour bar highlights the main clusters. (e/i/r)NSC: (endogenous/induced/reference) Neural Stem Cells, Gibco: EPSC neural induction with Gibco commercial protocol. N2B27: EPSC neural induction with bespoke published protocol[32]. Syn:syngeneic. Non Syn: non syngeneic.

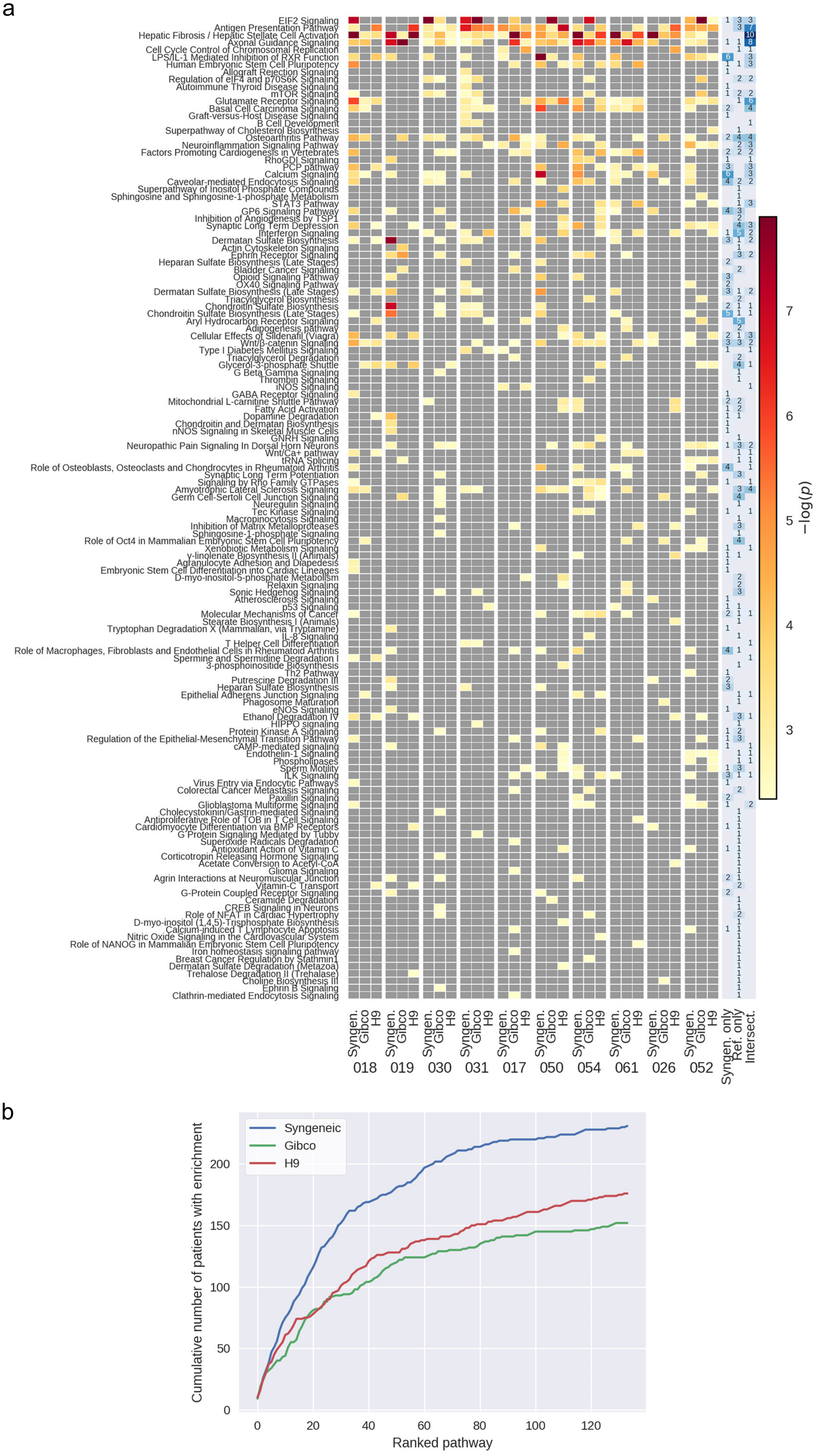
a
EIF2 Signaling
Antigen Presentation Pathway
Hepatic Fibrosis / Hepatic Stellate Cell Activation
Axonal Guidance Signaling
Cell Cycle Control of Chromosomal Replication
LPS/IL-1 Mediated Inhibition of RXR Function
Human Embryonic Stem Cell Pluripotency
Allograft Rejection Signaling
Regulation of eIF4 and p70S6K Signaling
Autoimmune Thyroid Disease Signaling
mTOR Signaling
Glutamate Receptor Signaling
Basal Cell Carcinoma Signaling
Graft-versus-Host Disease Signaling
B Cell Development
Superpathway of Cholesterol Biosynthesis
Osteoarthritis Pathway
Neuroinflammation Signaling Pathway
Factors Promoting Cardiogenesis in Vertebrates
RhoGDI Signaling
PCP pathway
Calcium Signaling
Caveolar-mediated Endocytosis Signaling
Superpathway of Inositol Phosphate Compounds
Sphingosine and Sphingosine-1-phosphate Metabolism
STAT3 Pathway
GP6 Signaling Pathway
Inhibition of Angiogenesis by TSP1
Synaptic Long Term Depression
Interferon Signaling
Dermatan Sulfate Biosynthesis
Actin Cytoskeleton Signaling
Ephrin Receptor Signaling
Heparan Sulfate Biosynthesis (Late Stages)
Bladder Cancer Signaling
Opioid Signaling Pathway
OX40 Signaling Pathway
Dermatan Sulfate Biosynthesis (Late Stages)
Triacylglycerol Biosynthesis
Chondroitin Sulfate Biosynthesis
Chondroitin Sulfate Biosynthesis (Late Stages)
Aryl Hydrocarbon Receptor Signaling
Adipogenesis pathway
Cellular Effects of Sildenafil (Viagra)
Wnt/β-catenin Signaling
Type I Diabetes Mellitus Signaling
Triacylglycerol Degradation
Glycerol-3-phosphate Shuttle
G Beta Gamma Signaling
Thrombin Signaling
iNOS Signaling
GABA Receptor Signaling
Mitochondrial L-carnitine Shuttle Pathway
Fatty Acid Activation
Dopamine Degradation
Chondroitin and Dermatan Biosynthesis
nNOS Signaling in Skeletal Muscle Cells
GNRH Signaling
Neuropathic Pain Signaling In Dorsal Horn Neurons
Wnt/Ca+ pathway
tRNA Splicing
Role of Osteoblasts, Osteoclasts and Chondrocytes in Rheumatoid Arthritis
Synaptic Long Term Potentiation
Signaling by Rho Family GTPases
Amyotrophic Lateral Sclerosis Signaling
Germ Cell-Sertoli Cell Junction Signaling
Neuregulin Signaling
Tec Kinase Signaling
Macropinocytosis Signaling
Inhibition of Matrix Metalloproteases
Sphingosine-1-phosphate Signaling
Role of Oct4 in Mammalian Embryonic Stem Cell Pluripotency
Xenobiotic Metabolism Signaling
γ-linolenate Biosynthesis II (Animals)
Agranulocyte Adhesion and Diapedesis
Embryonic Stem Cell Differentiation into Cardiac Lineages
D-myo-inositol-5-phosphate Metabolism
Relaxin Signaling
Sonic Hedgehog Signaling
Atherosclerosis Signaling
p53 Signaling
Molecular Mechanisms of Cancer
Stearate Biosynthesis I (Animals)
Tryptophan Degradation X (Mammalian, via Tryptamine)
IL-8 Signaling
T Helper Cell Differentiation
Role of Macrophages, Fibroblasts and Endothelial Cells in Rheumatoid Arthritis
Spermine and Spermidine Degradation I
3-phosphoinositide Biosynthesis
Th2 Pathway
Putrescine Degradation III
Heparan Sulfate Biosynthesis
Epithelial Adherens Junction Signaling
Phagosome Maturation
eNOS Signaling
Ethanol Degradation IV
HIPPO signaling
Protein Kinase A Signaling
Regulation of the Epithelial-Mesenchymal Transition Pathway
cAMP-mediated signaling
Endothelin-1 Signaling
Phospholipases
Sperm Motility
ILK Signaling
Virus Entry via Endocytic Pathways
Colorectal Cancer Metastasis Signaling
Paxillin Signaling
Glioblastoma Multiforme Signaling
Cholecystokinin/Gastrin-mediated Signaling
Antiproliferative Role of TOB in T Cell Signaling
Cardiomyocyte Differentiation via BMP Receptors
G Protein Signaling Mediated by Tubby
Superoxide Radicals Degradation
Antioxidant Action of Vitamin C
Corticotropin Releasing Hormone Signaling
Acetate Conversion to Acetyl-CoA
Glioma Signaling
Agrin Interactions at Neuromuscular Junction
Vitamin-C Transport
G-Protein Coupled Receptor Signaling
Ceramide Degradation
CREB Signaling in Neurons
Role of NFAT in Cardiac Hypertrophy
D-myo-inositol (1,4,5)-Trisphosphate Biosynthesis
Calcium-induced T Lymphocyte Apoptosis
Nitric Oxide Signaling in the Cardiovascular System
Role of NANOG in Mammalian Embryonic Stem Cell Pluripotency
Iron homeostasis signaling pathway
Breast Cancer Regulation by Stathmin1
Dermatan Sulfate Degradation (Metazoa)
Trehalose Degradation II (Trehalase)
Choline Biosynthesis III
Ephrin B Signaling
Clathrin-mediated Endocytosis Signaling
Syngen. Gibco H9
018 019 030 031 017 050 054 061 026 052
Syngen. only
Ref. only
Intersect.
−log(p)
3 4 5 6 7
b
Syngeneic
Gibco
H9
Cumulative number of patients with enrichment
Ranked pathway
0 50 100 150 200
0 20 40 60 80 100 120

Fig. S5: Comparative transcriptome analysis and signaling pathways deregulation between GIC, their matched-iNSC and non-syngeneic NSC.

a) Heatmap showing the IPA pathways identified as significantly enriched in the list of Differentially Expressed (DE) genes (GIC vs iNSC) of at least one patient. Shading indicates the significance of the enrichment. For clarity, we only show results for which the uncorrected *p* value is less than or equal to 0.005; grey values indicate a *p* value above this value. For each patient, we show results for the syngeneic GIC-iNSC comparison in addition to two comparisons of the same GIC line with reference NSC lines.

b) Syngeneic DE comparisons, carried out as described in Material and Methods section, identify more enriched IPA pathways than would be obtained by comparing the same GIC line with either of the two reference NSC lines. Each pathway is summarized by the number of patients exhibiting enrichment with each of the three NSC comparators ($p < 0.005$) and ranked based on the sum across all of them. This plot shows the cumulative total number of patients across the ranked pathway list for each comparator.

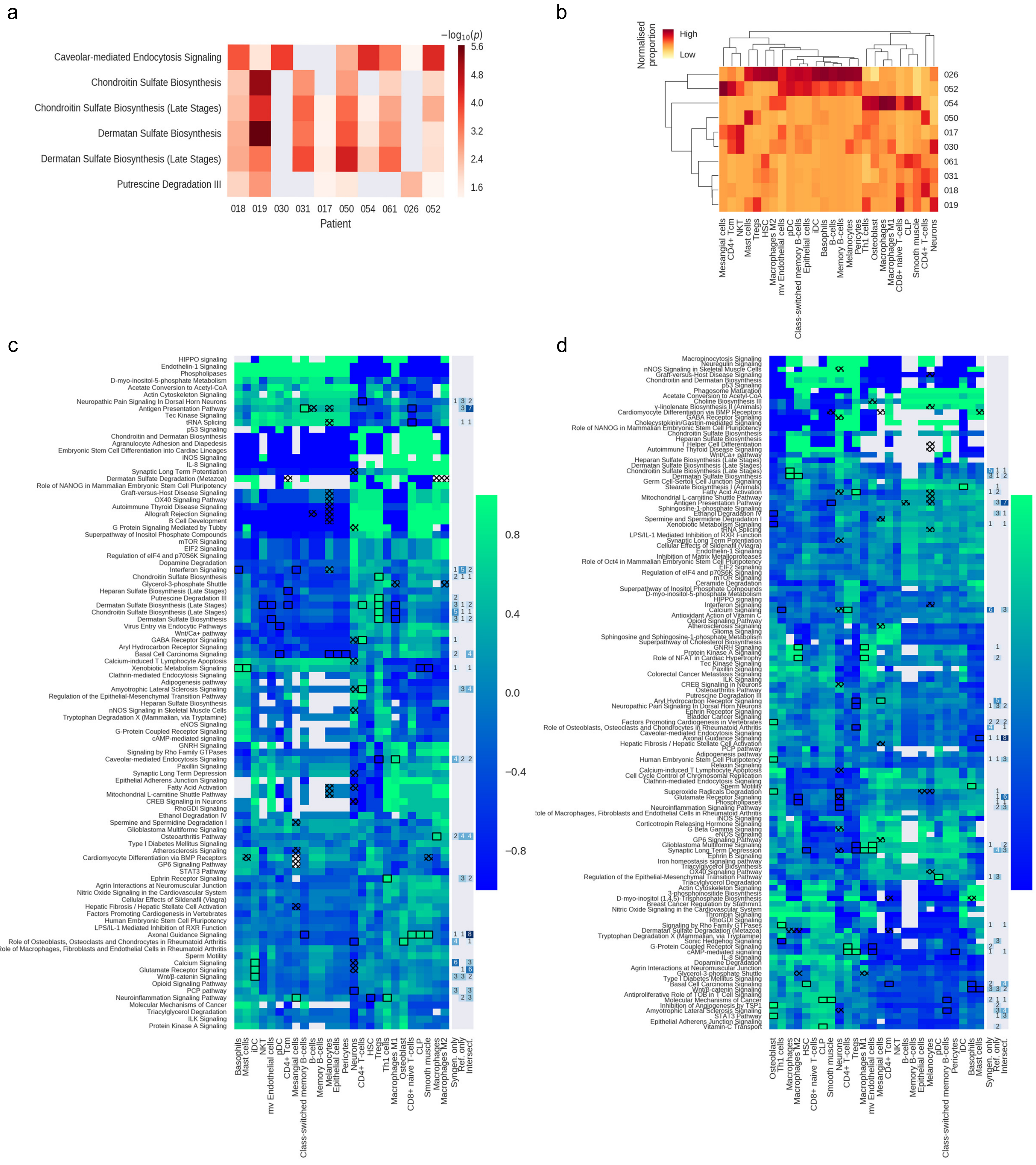
a
Caveolar-mediated Endocytosis Signaling
Chondroitin Sulfate Biosynthesis
Chondroitin Sulfate Biosynthesis (Late Stages)
Dermatan Sulfate Biosynthesis
Dermatan Sulfate Biosynthesis (Late Stages)
Putrescine Degradation III
018 019 030 031 017 050 054 061 026 052
Patient
$-\log_{10}(p)$
5.6
4.8
4.0
3.2
2.4
1.6
b
Normalised proportion
High
Low
026
052
054
050
017
030
061
031
018
019
Mesangial cells
CD4+ Tcm
NKT
Mast cells
Tregs
HSC
Macrophages M2
mv Endothelial cells
pDC
Class-switched memory B-cells
Epithelial cells
iDC
Basophils
B-cells
Memory B-cells
Melanocytes
Pericytes
Th1 cells
Osteoblast
Macrophages
Macrophages M1
CD8+ naive T-cells
CLP
Smooth muscle
CD4+ T-cells
Neurons
c
HIPPO signaling
Endothelin-1 Signaling
Phospholipases
D-myo-inositol-5-phosphate Metabolism
Acetate Conversion to Acetyl-CoA
Actin Cytoskeleton Signaling
Neuropathic Pain Signaling In Dorsal Horn Neurons
Antigen Presentation Pathway
Tec Kinase Signaling
tRNA Splicing
p53 Signaling
Chondroitin and Dermatan Biosynthesis
Agranulocyte Adhesion and Diapedesis
Embryonic Stem Cell Differentiation into Cardiac Lineages
iNOS Signaling
IL-8 Signaling
Synaptic Long Term Potentiation
Dermatan Sulfate Degradation (Metazoa)
Role of NANOG in Mammalian Embryonic Stem Cell Pluripotency
Graft-versus-Host Disease Signaling
OX40 Signaling Pathway
Autoimmune Thyroid Disease Signaling
Allograft Rejection Signaling
B Cell Development
G Protein Signaling Mediated by Tubby
Superpathway of Inositol Phosphate Compounds
mTOR Signaling
EIF2 Signaling
Regulation of eIF4 and p70S6K Signaling
Dopamine Degradation
Interferon Signaling
Chondroitin Sulfate Biosynthesis
Glycerol-3-phosphate Shuttle
Heparan Sulfate Biosynthesis (Late Stages)
Putrescine Degradation III
Dermatan Sulfate Biosynthesis (Late Stages)
Chondroitin Sulfate Biosynthesis (Late Stages)
Dermatan Sulfate Biosynthesis
Virus Entry via Endocytic Pathways
Wnt/Ca+ pathway
GABA Receptor Signaling
Aryl Hydrocarbon Receptor Signaling
Basal Cell Carcinoma Signaling
Calcium-induced T Lymphocyte Apoptosis
Xenobiotic Metabolism Signaling
Clathrin-mediated Endocytosis Signaling
Adipogenesis pathway
Amyotrophic Lateral Sclerosis Signaling
Regulation of the Epithelial-Mesenchymal Transition Pathway
Heparan Sulfate Biosynthesis
nNOS Signaling in Skeletal Muscle Cells
Tryptophan Degradation X (Mammalian, via Tryptamine)
eNOS Signaling
G-Protein Coupled Receptor Signaling
cAMP-mediated signaling
GNRH Signaling
Signaling by Rho Family GTPases
Caveolar-mediated Endocytosis Signaling
Paxillin Signaling
Synaptic Long Term Depression
Epithelial Adherens Junction Signaling
Fatty Acid Activation
Mitochondrial L-carnitine Shuttle Pathway
CREB Signaling in Neurons
RhoGDI Signaling
Ethanol Degradation IV
Spermine and Spermidine Degradation I
Glioblastoma Multiforme Signaling
Osteoarthritis Pathway
Type I Diabetes Mellitus Signaling
Atherosclerosis Signaling
Cardiomyocyte Differentiation via BMP Receptors
GP6 Signaling Pathway
STAT3 Pathway
Ephrin Receptor Signaling
Agrin Interactions at Neuromuscular Junction
Nitric Oxide Signaling in the Cardiovascular System
Cellular Effects of Sildenafil (Viagra)
Hepatic Fibrosis / Hepatic Stellate Cell Activation
Factors Promoting Cardiogenesis in Vertebrates
Human Embryonic Stem Cell Pluripotency
LPS/IL-1 Mediated Inhibition of RXR Function
Axonal Guidance Signaling
Role of Osteoblasts, Osteoclasts and Chondrocytes in Rheumatoid Arthritis
Role of Macrophages, Fibroblasts and Endothelial Cells in Rheumatoid Arthritis
Sperm Motility
Calcium Signaling
Glutamate Receptor Signaling
Wnt/β-catenin Signaling
Opioid Signaling Pathway
PCP pathway
Neuroinflammation Signaling Pathway
Molecular Mechanisms of Cancer
Triacylglycerol Degradation
ILK Signaling
Protein Kinase A Signaling
Basophils
Mast cells
iDC
NKT
mv Endothelial cells
pDC
CD4+ Tcm
Mesangial cells
Class-switched memory B-cells
B-cells
Memory B-cells
Melanocytes
Epithelial cells
Pericytes
Neurons
CD4+ T-cells
HSC
Tregs
Th1 cells
Macrophages M1
Osteoblast
CD8+ naive T-cells
CLP
Smooth muscle
Macrophages
Macrophages M2
Syngen. only
Ref. only
Intersect.
0.8
0.4
0.0
−0.4
−0.8
d
Macropinocytosis Signaling
Neuregulin Signaling
nNOS Signaling in Skeletal Muscle Cells
Graft-versus-Host Disease Signaling
Chondroitin and Dermatan Biosynthesis
p53 Signaling
Phagosome Maturation
Acetate Conversion to Acetyl-CoA
Choline Biosynthesis III
γ-linolenate Biosynthesis II (Animals)
Cardiomyocyte Differentiation via BMP Receptors
GABA Receptor Signaling
Cholecystokinin/Gastrin-mediated Signaling
Role of NANOG in Mammalian Embryonic Stem Cell Pluripotency
Chondroitin Sulfate Biosynthesis
Heparan Sulfate Biosynthesis
T Helper Cell Differentiation
Autoimmune Thyroid Disease Signaling
Wnt/Ca+ pathway
Heparan Sulfate Biosynthesis (Late Stages)
Dermatan Sulfate Biosynthesis (Late Stages)
Chondroitin Sulfate Biosynthesis (Late Stages)
Dermatan Sulfate Biosynthesis
Germ Cell-Sertoli Cell Junction Signaling
Stearate Biosynthesis I (Animals)
Fatty Acid Activation
Mitochondrial L-carnitine Shuttle Pathway
Antigen Presentation Pathway
Sphingosine-1-phosphate Signaling
Ethanol Degradation IV
Spermine and Spermidine Degradation I
Xenobiotic Metabolism Signaling
tRNA Splicing
LPS/IL-1 Mediated Inhibition of RXR Function
Synaptic Long Term Potentiation
Cellular Effects of Sildenafil (Viagra)
Endothelin-1 Signaling
Inhibition of Matrix Metalloproteases
Role of Oct4 in Mammalian Embryonic Stem Cell Pluripotency
EIF2 Signaling
Regulation of eIF4 and p70S6K Signaling
mTOR Signaling
Ceramide Degradation
Superpathway of Inositol Phosphate Compounds
D-myo-inositol-5-phosphate Metabolism
HIPPO signaling
Interferon Signaling
Calcium Signaling
Antioxidant Action of Vitamin C
Opioid Signaling Pathway
Atherosclerosis Signaling
Glioma Signaling
Sphingosine and Sphingosine-1-phosphate Metabolism
Superpathway of Cholesterol Biosynthesis
GNRH Signaling
Protein Kinase A Signaling
Role of NFAT in Cardiac Hypertrophy
Tec Kinase Signaling
Paxillin Signaling
Colorectal Cancer Metastasis Signaling
ILK Signaling
CREB Signaling in Neurons
Osteoarthritis Pathway
Putrescine Degradation III
Aryl Hydrocarbon Receptor Signaling
Neuropathic Pain Signaling In Dorsal Horn Neurons
Ephrin Receptor Signaling
Bladder Cancer Signaling
Factors Promoting Cardiogenesis in Vertebrates
Role of Osteoblasts, Osteoclasts and Chondrocytes in Rheumatoid Arthritis
Caveolar-mediated Endocytosis Signaling
Axonal Guidance Signaling
Hepatic Fibrosis / Hepatic Stellate Cell Activation
PCP pathway
Adipogenesis pathway
Human Embryonic Stem Cell Pluripotency
Relaxin Signaling
Calcium-induced T Lymphocyte Apoptosis
Cell Cycle Control of Chromosomal Replication
Clathrin-mediated Endocytosis Signaling
Sperm Motility
Superoxide Radicals Degradation
Glutamate Receptor Signaling
Phospholipases
Neuroinflammation Signaling Pathway
Role of Macrophages, Fibroblasts and Endothelial Cells in Rheumatoid Arthritis
iNOS Signaling
Corticotropin Releasing Hormone Signaling
G Beta Gamma Signaling
eNOS Signaling
GP6 Signaling Pathway
Glioblastoma Multiforme Signaling
Synaptic Long Term Depression
Ephrin B Signaling
Iron homeostasis signaling pathway
Triacylglycerol Biosynthesis
OX40 Signaling Pathway
Regulation of the Epithelial-Mesenchymal Transition Pathway
Triacylglycerol Degradation
Actin Cytoskeleton Signaling
3-phosphoinositide Biosynthesis
D-myo-inositol (1,4,5)-Trisphosphate Biosynthesis
Breast Cancer Regulation by Stathmin1
Nitric Oxide Signaling in the Cardiovascular System
Thrombin Signaling
RhoGDI Signaling
Signaling by Rho Family GTPases
Dermatan Sulfate Degradation (Metazoa)
Tryptophan Degradation X (Mammalian, via Tryptamine)
Sonic Hedgehog Signaling
G-Protein Coupled Receptor Signaling
cAMP-mediated signaling
IL-8 Signaling
Dopamine Degradation
Agrin Interactions at Neuromuscular Junction
Glycerol-3-phosphate Shuttle
Type I Diabetes Mellitus Signaling
Basal Cell Carcinoma Signaling
Wnt/β-catenin Signaling
Antiproliferative Role of TOB in T Cell Signaling
Molecular Mechanisms of Cancer
Inhibition of Angiogenesis by TSP1
Amyotrophic Lateral Sclerosis Signaling
STAT3 Pathway
Epithelial Adherens Junction Signaling
Vitamin-C Transport
Osteoblast
Th1 cells
Macrophages
Macrophages M2
HSC
CD8+ naive T-cells
CLP
Smooth muscle
Neurons
CD4+ T-cells
Tregs
Macrophages M1
mv Endothelial cells
Mesangial cells
CD4+ Tcm
NKT
B-cells
Memory B-cells
Epithelial cells
Melanocytes
pDC
Class-switched memory B-cells
Pericytes
iDC
Basophils
Mast cells
Syngen. only
Ref. only
Intersect.

Fig. S6: Integration of inferred cell type proportion of the tumour bulk and pathway enrichment in hGIC vs iNSC.

a) Heatmap showing the Spearman rank correlation across patients between pathway enrichment in the GIC culture relative to matched iNSC, expressed as –log10(*p*), and estimated cell type composition in the corresponding GBM bulk tissue sample (See M&M for full details). 6 pathways of interest are included. Grey shading indicates no statistical significance (unadjusted *p* value>0.05).

b) Heatmap and dendrograms showing the estimated proportion of different cell types (columns) across patients (rows), normalised by column.

c) Heatmap showing the Spearman rank correlation across patients between pathway enrichment in the GIC culture relative to matched iNSC, expressed as –log10(*p*), and estimated cell type composition in the corresponding GBM bulk tissue sample (see M&M for full details). Blank areas indicate pathways for which too few patients have p values defined. Highlighted cells show the combinations for which the correlation coefficient is statistically significant (unadjusted *p* value<0.05). Hatched cells are those combinations in which the overlap between the cell subtype signature and the pathway genes is large. Only pathways enriched in at least one syngeneic comparison (unadjusted *p* value<0.05) are shown. (Right panel) Numbers of patient comparisons exhibiting a statistically significant enrichment (unadjusted *p* value<0.005) in the syngeneic and reference DE comparisons. Results correspond to regions on a Venn diagram (left to right: only in the syngeneic comparison, only in the reference comparison, in both).

d) As for (c), but selecting pathways enriched in at least one reference comparison (unadjusted *p* value<0.05).

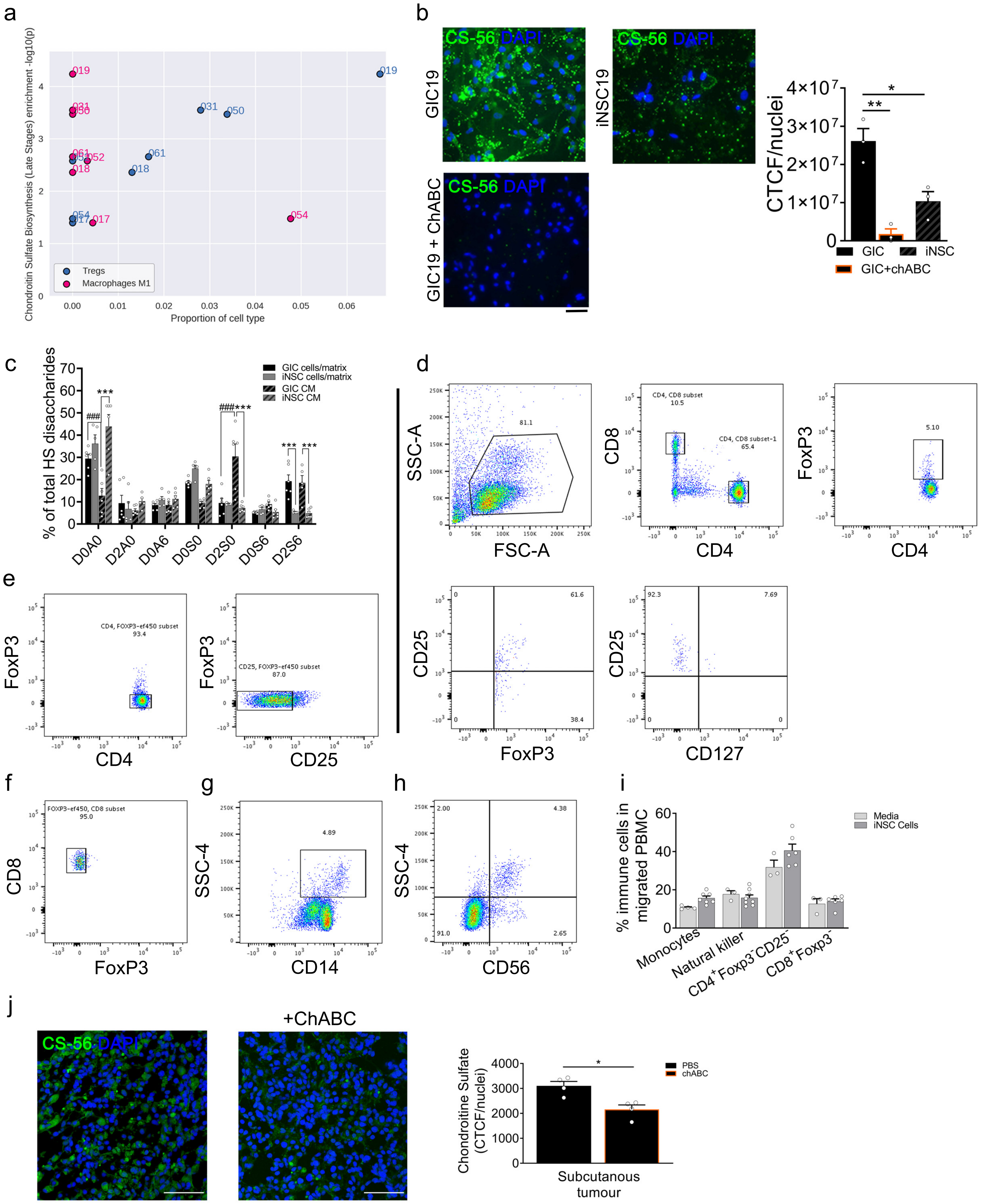
a
Chondroitin Sulfate Biosynthesis (Late Stages) enrichment -log10(p)
Proportion of cell type
Tregs
Macrophages M1
b
CS-56 DAPI
GIC19
iNSC19
GIC19 + ChABC
CTCF/nuclei
GIC
iNSC
GIC+chABC
c
% of total HS disaccharides
GIC cells/matrix
iNSC cells/matrix
GIC CM
iNSC CM
D0A0
D2A0
D0A6
D0S0
D2S0
D0S6
D2S6
d
SSC-A
FSC-A
CD4, CD8 subset
CD4, CD8 subset-1
CD8
CD4
FoxP3
CD25
CD127
e
CD4, FOXP3-ef450 subset
CD25, FOXP3-ef450 subset
f
FOXP3-ef450, CD8 subset
g
SSC-4
CD14
h
CD56
i
% immune cells in migrated PBMC
Media
iNSC Cells
Monocytes
Natural killer
CD4+Foxp3-CD25-
CD8+Foxp3-
j
+ChABC
Chondroitine Sulfate (CTCF/nuclei)
PBS
chABC
Subcutanous tumour

Fig. S7: GAG analysis and modulation of immune cells migration by GIC as compared to iNSC.

a) Scatterplot showing the correlation between estimated cell type proportion in bulk GBM tissue (from FFPE blocks) and the extent to which the Differentially Expressed (DE) genes in the GIC vs iNSC comparison are enriched for the IPA pathway 'Chondroitin Sulfate Biosynthesis (Late Stages)'. Two different cell types are shown for which the correlation is statistically significant (Fig.S6C).

b) Representative images of Chondroitin Sulfate (CS) immunostaining (clone CS-56) in GIC19, GIC19+chondroitinase ABC (chABC) treatment and iNSC19 and quantification. Results are expressed in CTCF (Corrected Total Cell Fluorescence). Scale bar is 20µm. Results are an average from two patients 19 and 31 (n=2, chABC treatment experiments have been repeated 3 times, one-way ANOVA, *p* value ((GIC vs chABC)=0,0012, *p* value (GIC vs iNSC)=0,0105).

c) Disaccharide composition of Heparan Sulfate (HS) isolated from GIC (black bars) and iNSC (grey bars) cell extracts (plain bars) and from conditioned media (hatched bars) analysed by RP-HPLC. D0A0: HexA-GlcNAc; D2A0: HexA(2S)-GlcNAc; D0A6: HexA-GlcNAc(6S); D0S0: HexA-GlcNS; D2S0: HexA(2S)-GlcNS; D0S6, HexA-GlcNS(6S); D2S6: HexA(2S)-GlcNS(6S). Results are an average of two GIC/iNSC pairs, 19 and 31, experiment has been repeated 3 times, two-way ANOVA, D0A0: *p* value (GIC cells/matrix vs GIC CM)<0,0001, *p* value (GIC CM vs GIC CM)<0,0001; D2S0: *p* value (GIC cells/matrix vs GIC CM)<0,0001, *p* value (GIC CM vs GIC CM)<0,0001; D2S6: *p* value (GIC cells/matrix vs iNSC cells/matrix)=0,0006, *p* value (GIC CM vs iNSC CM)=0,0007.

d-h) Flow cytometry plots representing gating to assess percentage of Tregs ($CD4^+CD8^-Foxp3^+CD25^+CD127^-$) (D), $CD4^+Foxp3^-CD25^-$ (E) and $CD8^+Foxp3^-$ (F) T cells, monocytes ($CD14^+$) (G) natural killer cells ($CD56^+$) (H) in the Peripheral Blood Mononuclear Cell (PBMC) migrated population of Figure3 E-F.

i) Percentages of migrated PBMC immune cells monocytes (CD14+), natural killer (CD56+), CD4+Foxp3-Cd25- and CD8+Foxp3- T cells in presence of media only (light grey bars) or iNSC (dark grey bars) after 4 hours incubation. Results are an average from two patients 19 and 31 (n=2, 4 technical replicas, repeated 4 independent times, one-way ANOVA, *p* value (Monocytes)=0,7011, *p* value ($CD4^+Foxp3^-CD25^-$)=0,1814.

j) Representative images of CS immunostaining in subcutaneous GIC19-derived xenografts treated with vehicle or chABC and quantification. Results are expressed in CTCF (Corrected Total Cell Fluorescence). Scale bar is 50µm. N=4 tumours per condition, two tailed t test, p value=0,0101.

All graphs report mean ±SEM. Statistical significance for all panels **p*≤0.05, ***p*≤0.01, ****p*≤0.001. Source data are provided in the source data file.

a

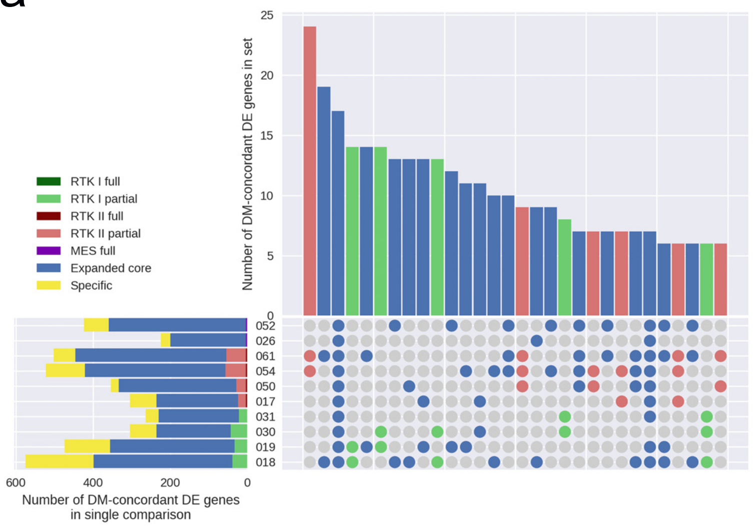

b

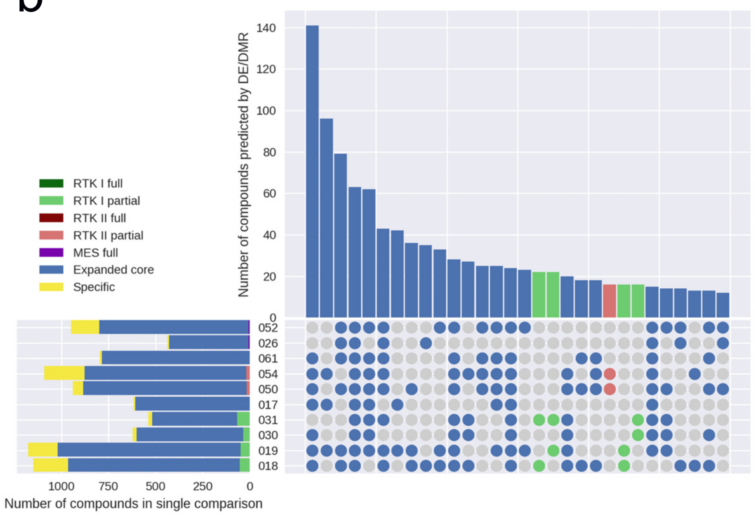

c

**Gene**	***PTGER4***	***NTRK2***	***ALDH3B1***
**Signature**	High meth/Low exp	Low meth/High exp	Low meth/High exp
**% of GICs**	34%	21%	22.50%

Fig. S8: Characterisation of patient-specific drug sensitivity.

a) Upset plot showing the number of concordant Differentially Expressed/Differentially Methylated Regions (DE/DMRs) (features) shared across different combinations of patients. The lower left panel quantifies the total number of features independently in each patient; shading highlights the proportions of these that are specific to that patient or shared only with patients from the same methylation-derived subgroup. 'Full 'refers to features shared by all patients assigned to a subgroup; 'partial 'refers to those shared by only some of those patients. 'Expanded core 'refers to features that are identified in >1 patient line and across known subgroups. The right-hand panel quantifies the number of features in different combinations of patients, in descending order. For example, patients 54 and 61 (both in the RTK II subgroup) share 24 concordant DE/DMRs. MES: Mesenchymal. RTKI and II: Receptor Tyrosin Kinase subgroup I and II.

b) Upset plot showing the number of drug compounds identified based on the concordant DE/DMRs in each patient. The interpretation of this plot is similar to Fig.S8A.

c) Table showing proportion of GIC within the Human Glioblastoma Cell Culture (HGCC) cohort with high methylation and low expression for *PTGER4* and low methylation and high expression for *NTRK2* and *ALDH3B1* as compared to cohort averages.

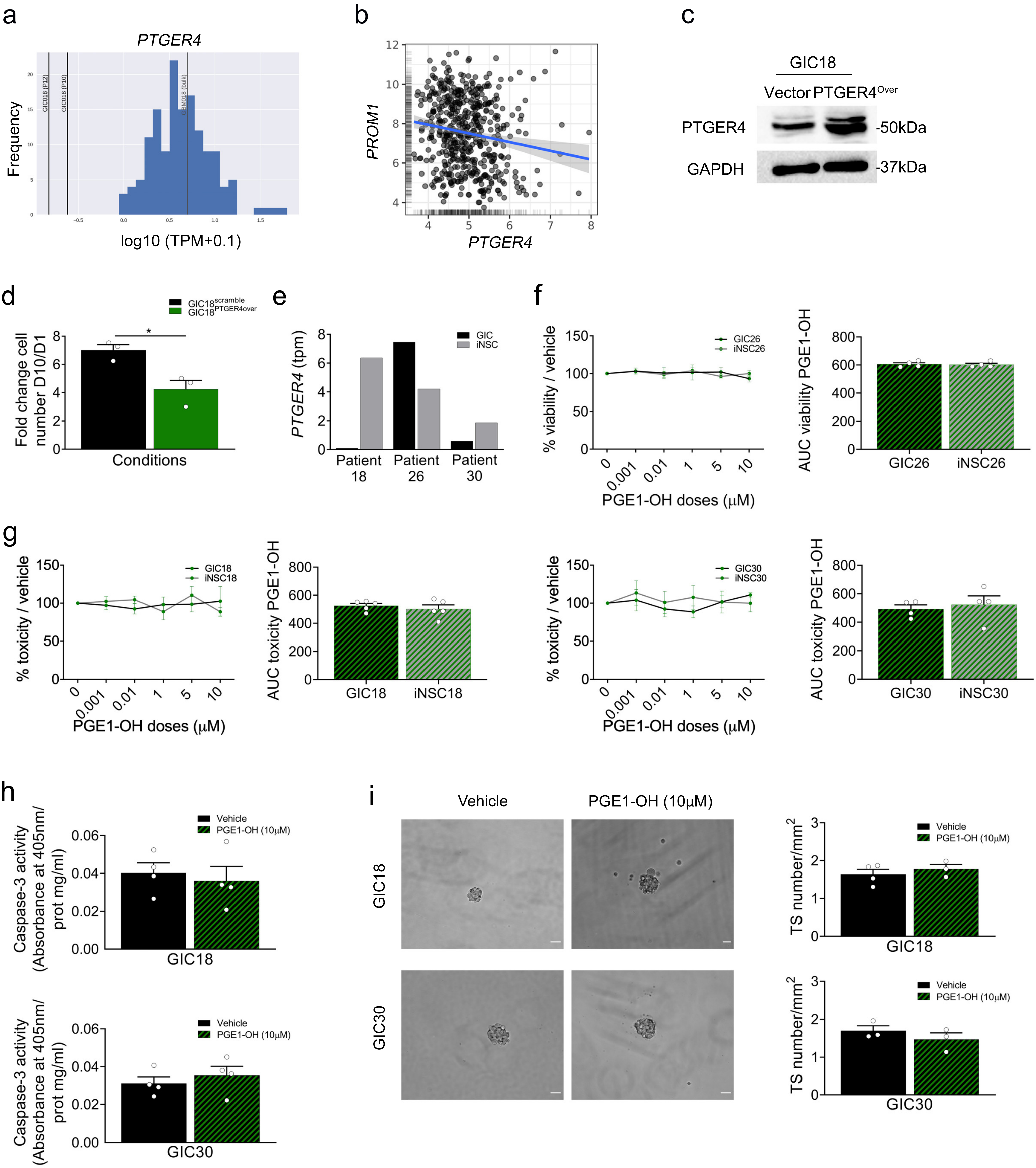

a
PTGER4
Frequency
GIC018 (P12)
GIC018 (P10)
GBM018 (bulk)
log10 (TPM+0.1)
b
PROM1
PTGER4
c
GIC18
Vector
PTGER4Over
PTGER4
-50kDa
GAPDH
-37kDa
d
GIC18scramble
GIC18PTGER4over
Fold change cell number D10/D1
Conditions
e
GIC
iNSC
PTGER4 (tpm)
Patient 18
Patient 26
Patient 30
f
GIC26
iNSC26
% viability / vehicle
PGE1-OH doses (μM)
AUC viability PGE1-OH
GIC26
iNSC26
g
GIC18
iNSC18
% toxicity / vehicle
PGE1-OH doses (μM)
AUC toxicity PGE1-OH
GIC18
iNSC18
GIC30
iNSC30
% toxicity / vehicle
PGE1-OH doses (μM)
AUC toxicity PGE1-OH
GIC30
iNSC30
h
Vehicle
PGE1-OH (10μM)
Caspase-3 activity (Absorbance at 405nm/ prot mg/ml)
GIC18
Vehicle
PGE1-OH (10μM)
Caspase-3 activity (Absorbance at 405nm/ prot mg/ml)
GIC30
i
Vehicle
PGE1-OH (10μM)
GIC18
GIC30
Vehicle
PGE1-OH (10μM)
TS number/mm2
GIC18
Vehicle
PGE1-OH (10μM)
TS number/mm2
GIC30

Fig. S9: PTGER4 in GBM and GIC and characterization of PGE1-OH treatment in 2D cultures.

a) Expression of *PTGER4* in the GIC18 line (two passages, black lines) relative to the bulk tissue (grey line) and the TCGA GBM cohort (blue histogram; includes IDH wildtype cases only).
b) Correlation analysis of *PTGER4* and *PROM1* in GBM (TCGA, *p*value <0.01 and correlation coefficient of -0.14 (Pearson coefficient). Obtained from http://gliovis.bioinfo.cnio.es/
c) Western blot showing expression level of PTGER4 in GIC18 (scramble control) and GIC18 overexpressing PTGER4. GAPDH is used as housekeeping gene. Experiments have been performed 3 independent times with consistent results.
d) Proliferation assay with results represented as fold change of cell number at day 10 day 1 after plating in GIC18 transduced with empty plasmid (black dots) and *PTGER4* overexpressing plasmid (green dots) (n=3 repetition of experiment, two tailed *t* test, *p* value=0,0195).
e) mRNA expression of PTGER4 (tpm from RNA sequencing analysis) in iNSC (grey bars) and GIC (black bars) for patient 18, 26 and 30.
f) PGE1-OH treatment of GIC (black curves) and iNSC (grey curves) of patient 26 with increasing doses (1nM to 10µM) (green dots). Results are represented as percentage of cell viability compared to the vehicle, measured at end point; area under the curve (AUC) was calculated from percentages of viability (n=4 times, two tailed t test, p value=0,8541).
g) PGE1-OH treatment of GIC (black curves) and iNSC (grey curves) of patient 18 (left) and 30 (right) with increasing doses (1nM to 10µM) (green dots). Results are represented as percentage of cytotoxicity on the vehicle, measured at end point; area under the curve (AUC) calculated from percentages of toxicity (GIC18: n=6 and GIC30: n=4 repetition of experiment, two tailed *t* test, *p* value (patient 18)=0,5091, *p* value (patient 30)=0,6605).
h) Apoptosis, as assessed by Caspase 3 activity, measured on vehicle (black bars) or PGE1-OH 10µM (green hatched pattern) treated GIC18 (top) and 30 (bottom) after 4 days of treatment (n=3,, two tailed *t* test, *p* value (patient 18)=0,6733, *p* value (patient 30)=0,4979).
i) Representative images of tumour spheres (TS) arising from GIC18 (top) and 30 (bottom)
cultured in non-adherent condition after 4 days of treatments with vehicle (left) or PGE1-OH 10 µM (right) (scale bar is 20µm) and quantification of TS number per area (right) (n=3, two tailed *t* test , *p* value (patient 18)=0,4829 , *p* value (patient 30)=0,3483).
All graphs report mean ±SEM. Statistical significance for all panels **p*≤0.05, ***p*≤0.01, ****p*≤0.001. Source data are provided in the source data file.

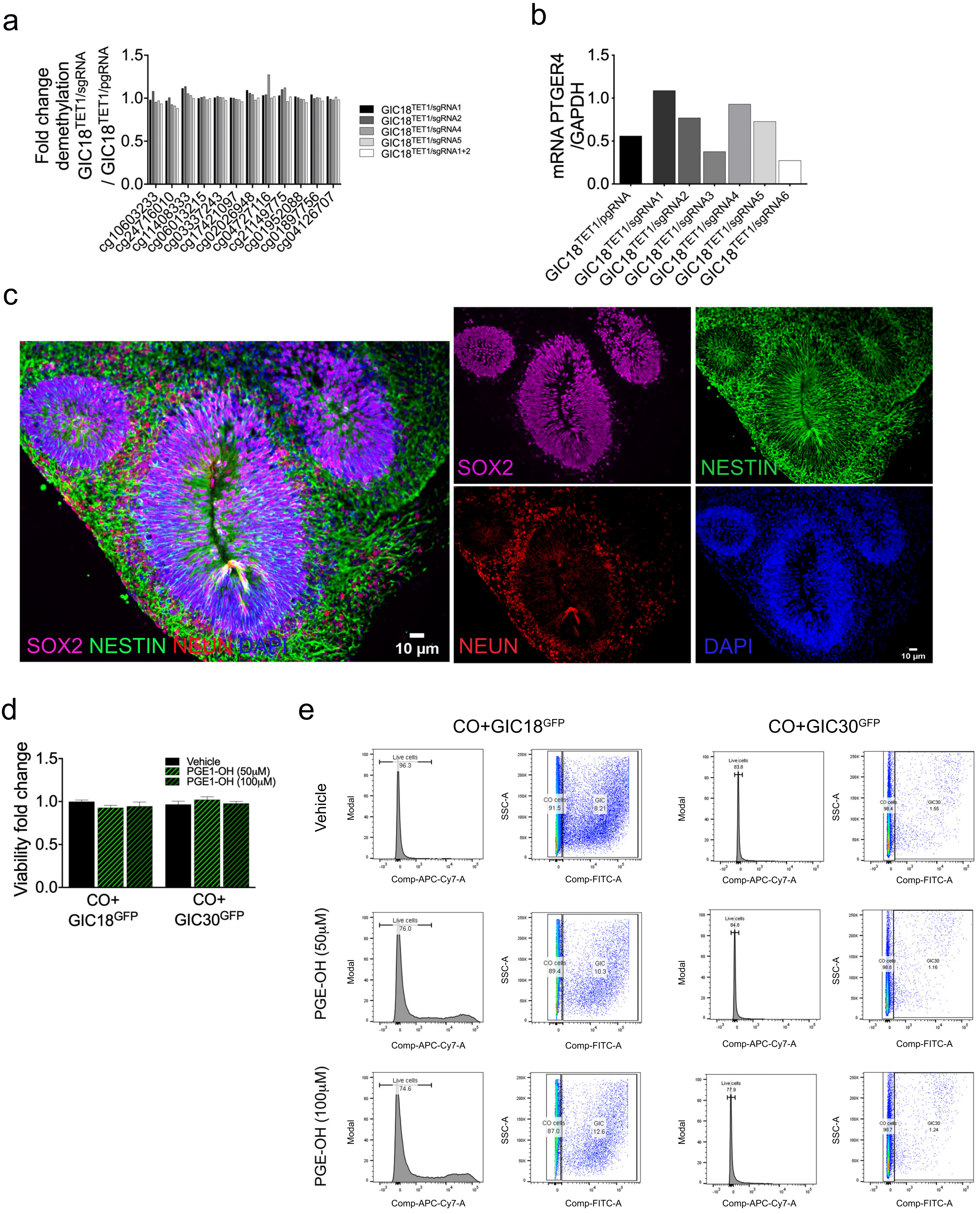

a
Fold change demethylation GIC18TET1/sgRNA / GIC18TET1/pgRNA
1.5
1.0
0.5
0.0
cg10603233
cg24716010
cg11408333
cg06013215
cg03337243
cg17421097
cg02026948
cg04727116
cg21149775
cg01952088
cg01897756
cg04126707
GIC18TET1/sgRNA1
GIC18TET1/sgRNA2
GIC18TET1/sgRNA4
GIC18TET1/sgRNA5
GIC18TET1/sgRNA1+2
b
mRNA PTGER4 /GAPDH
GIC18TET1/pgRNA
GIC18TET1/sgRNA1
GIC18TET1/sgRNA2
GIC18TET1/sgRNA3
GIC18TET1/sgRNA4
GIC18TET1/sgRNA5
GIC18TET1/sgRNA6
c
SOX2 NESTIN NEUN DAPI
10 µm
SOX2
NESTIN
NEUN
DAPI
10 µm
d
Viability fold change
Vehicle
PGE1-OH (50µM)
PGE1-OH (100µM)
CO+ GIC18GFP
CO+ GIC30GFP
e
CO+GIC18GFP
CO+GIC30GFP
Vehicle
PGE-OH (50µM)
PGE-OH (100µM)
Modal
SSC-A
Comp-APC-Cy7-A
Comp-FITC-A
Live cells 96.3
CO cells 91.5
GIC 8.21
Live cells 83.8
CO cells 98.4
GIC30 1.55
Live cells 76.0
CO cells 89.4
GIC 10.3
Live cells 84.8
CO cells 98.8
GIC30 1.16
Live cells 74.6
CO cells 87.0
GIC 12.6
Live cells 77.9
CO cells 98.7
GIC30 1.24

Fig. S10: Epigenetic editing of PTGER4 and CO characterization.

a) Fold change demethylation of selected guides of $GIC18^{TET1}$ at different CpG islands within the hypermethylated region. Location of the CpG dinucleotides on the Infinium MethylationEPIC array is shown on the x axis.

b) *PTGER4* mRNA expression level in $GIC18^{TET1/pgRNA}$ (control line, black bar) and GIC18 infected with dCas9-*TET1* and the 6 guides RNA (grey bars).

c) Immunofluorescence staining for SOX2 (purple), NESTIN (green) and NEUN (red) in 37days old CO (representative images, DAPI is used as counterstaining). Scale bar is 10 µm. Staining have been performed on CO derived from 2 patients with consistent results.

d) Viability of total cells of the SYNGLICO model, assessed as percentages of negative cells for Zombie NIR^{TM} after 4 days of treatment with vehicle or PGE1-OH (50µM, 100µM); COGIC18 vehicle: n=12, PGE1-OH 50µM : n=11, PGE1-OH 100µM: n=10; COGIC19 vehicle: n=9, PGE1-OH 50µM: n=12 and PGE1-OH: n=11 SYNGLICO per group, one way ANOVA, Patient 18: *p* value (Veh vs PGE1-OH 50µM)=0,2596, *p* value (Veh vs PGE1-OH 100µM)=0,4683

e) Representative FACS plots of overall viability of the SYNGLICO (Cerebral Organoids (CO)+$GIC18^{GFP}$) (left) or control patient CO+$GIC30^{GFP}$ (right) analysed 4 days post-treatment with vehicle or PGE1-OH (50µM, 100µM) after gating for Zombie NIR^{TM} negative cells (live cells) shown on plots as histogram of Comp-APC-Cy7-A, and total GFP population after gating for FITC, from which the GFP Mean Fluorescence Intensity (MFI) was calculated.

All graphs report mean ±SEM. Statistical significance for all panels *p≤0.05, **p≤0.01, ***p≤0.001. Source data are provided in the source data file.

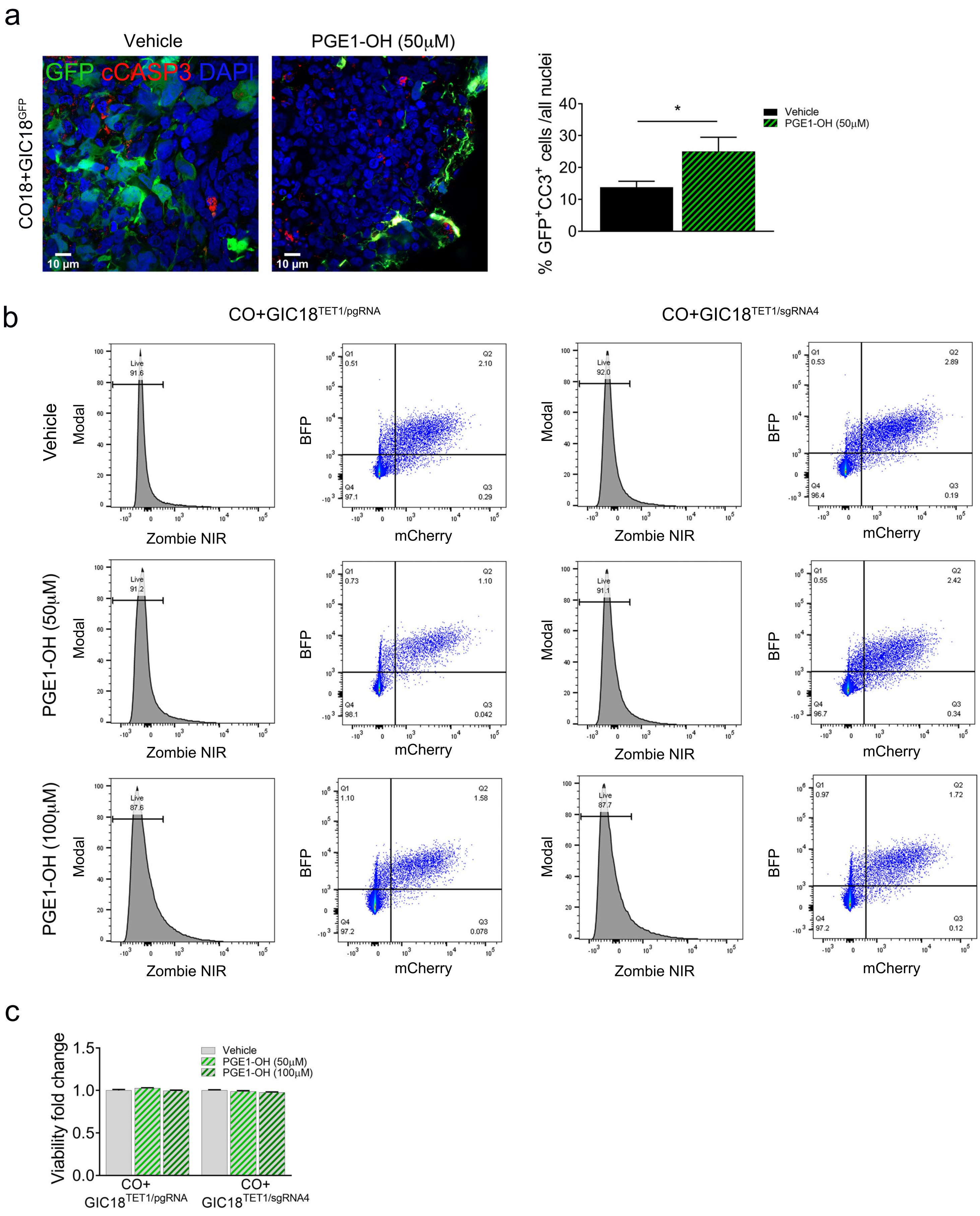
a
Vehicle
PGE1-OH (50μM)
GFP cCASP3 DAPI
CO18+GIC18GFP
10 μm
10 μm
% GFP+CC3+ cells /all nuclei
*
Vehicle
PGE1-OH (50μM)
b
CO+GIC18TET1/pgRNA
CO+GIC18TET1/sgRNA4
Vehicle
PGE1-OH (50μM)
PGE1-OH (100μM)
Modal
BFP
Zombie NIR
mCherry
c
Viability fold change
Vehicle
PGE1-OH (50μM)
PGE1-OH (100μM)
CO+ GIC18TET1/pgRNA
CO+ GIC18TET1/sgRNA4

Fig. S11: SYNGLICO characterization and analysis of the effect of PGE1-OH treatment on viability and CO^{GFP+} MFI.

a) Representative images of GFP and cleaved Caspase 3 (cCasp3) IF of SYNGLICO (Cerebral Organoids (CO)+GIC18^{GFP}) after 4 days of treatment with vehicle or PGE1-OH (50µM). Scale bar is 10µm. Right panel shows quantification of GFP and cCasp3 double positive cells (n=3 biological replicates, Vehicle: n=14 fields, PGE1-OH 50µM: n=13 fields, two tailed *t* test, *p* value=0,0250.

b) Representative FACS plots of total COGIC18$^{TET1/pgRNA}$ (left) and COGIC18$^{TET1/sgRNA4}$ (right) processed and analysed 4 days post-treatment with vehicle or PGE1-OH (50µM, 100µM). Overall viability of the SYNGLICO was assessed after gating for Zombie NIR^{TM} negative cells (live cells) shown on plots as histogram of Comp-APC-Cy7-A. BFP/mCherry double positive cell populations were gated (Q2 population on plots), from which the MFI of BFP was calculated.

c) Viability of GIC18$^{TET1/pgRNA}$ and GIC18$^{TET1/sgRNA4}$ assessed as percentages of negative cells for Zombie NIR^{TM} after 4 days of treatment with vehicle or PGE1-OH (50µM, 100µM); n=6 SYNGLICO per group, one-way ANOVA, pgRNA: *p* value (Veh vs PGE1-OH 50µM)=0,1191, *p* value (Veh vs PGE1-OH 100µM)=0,9373, sgRNA: *p* value (Veh vs PGE1-OH 50µM)=0,6403, *p* value (Veh vs PGE1-OH 100µM)=0,1497.

All graphs report mean ±SEM. Statistical significance for all panels *$p \leq 0.05$, **$p \leq 0.01$, ***$p \leq 0.001$. Source data are provided in the source data file.

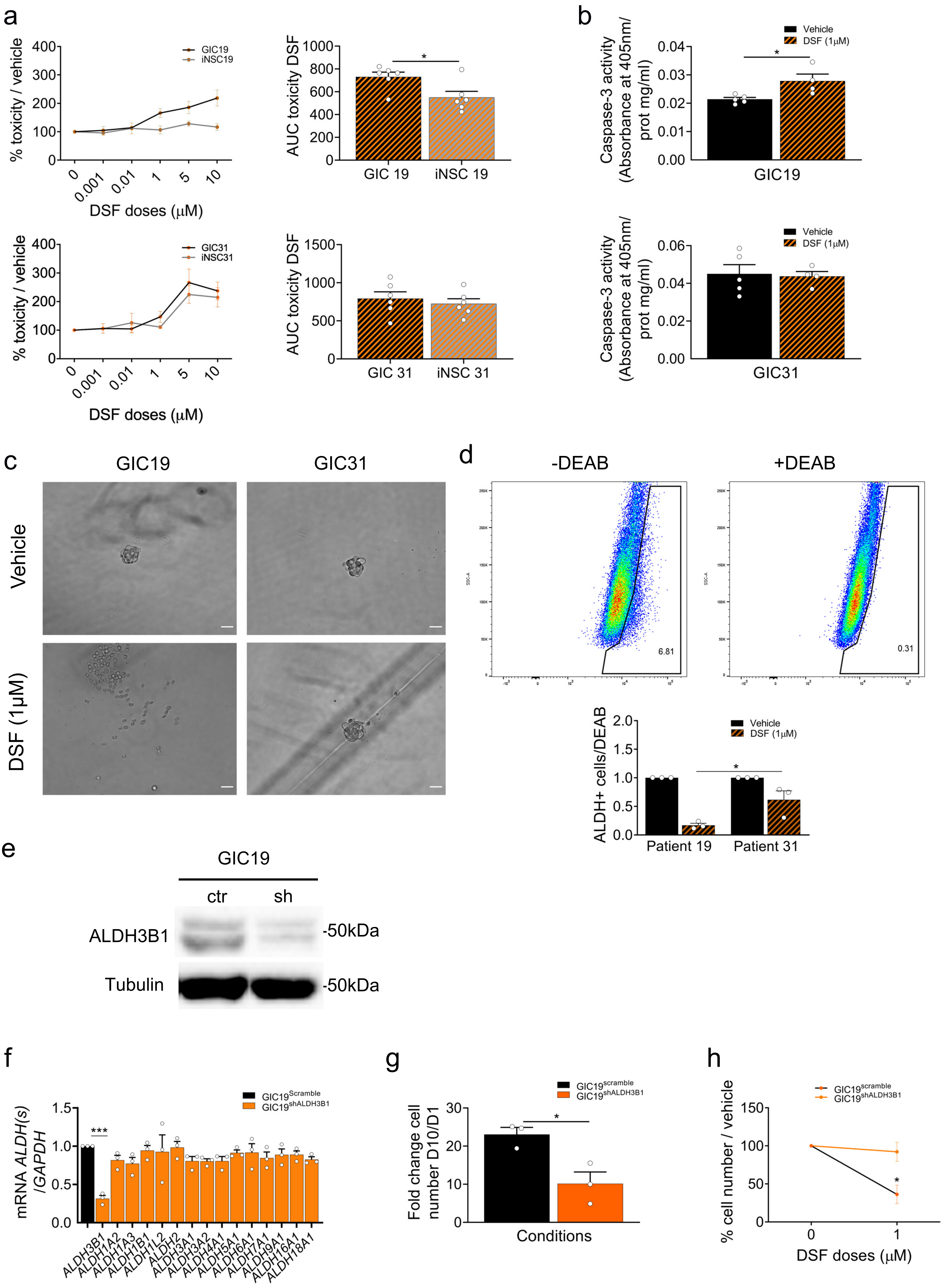
a
% toxicity / vehicle
GIC19
iNSC19
DSF doses (μM)
AUC toxicity DSF
GIC 19
iNSC 19
GIC31
iNSC31
GIC 31
iNSC 31
b
Caspase-3 activity (Absorbance at 405nm/ prot mg/ml)
Vehicle
DSF (1μM)
GIC19
GIC31
c
GIC19
GIC31
Vehicle
DSF (1μM)
d
-DEAB
+DEAB
6.81
0.31
ALDH+ cells/DEAB
Vehicle
DSF (1μM)
Patient 19
Patient 31
e
GIC19
ctr
sh
ALDH3B1
-50kDa
Tubulin
-50kDa
f
mRNA ALDH(s) /GAPDH
GIC19Scramble
GIC19shALDH3B1
***
ALDH3B1
ALDH1A2
ALDH1A3
ALDH1B1
ALDH1L2
ALDH2
ALDH3A1
ALDH3A2
ALDH4A1
ALDH5A1
ALDH6A1
ALDH7A1
ALDH9A1
ALDH16A1
ALDH18A1
g
Fold change cell number D10/D1
GIC19scramble
GIC19shALDH3B1
Conditions
h
% cell number / vehicle
GIC19scramble
GIC19shALDH3B1
DSF doses (μM)

Fig. S12: ***ALDH3B1/DSF treatment characterisation in 2D culture.***

a) Drug treatment on GIC (black curves) and iNSC (grey curves) of patient 19 (left) and 31 (right) with doses ranging from 1nM to 10µM of DSF (orange dots). Results are expressed in percentage of cytotoxicity on the vehicle and were measured at end point, area under the curve (AUC) calculated from percentages of toxicity (n=6 times, two tailed *t* test, *p* value (patient 19)=0,0247, *p* value (patient 31)=0,5496).

b) Apoptosis as assessed by Caspase 3 activity, measured after 4 days of treatment with vehicle (black bars) or DSF 1µM (orange hatched pattern) in GIC19 (top) and control GIC31 (bottom) (n=3 repetitions of experiment, two tailed *t* test *p* value (patient 19)=0,0247, *p* value (patient 31)=0,8313).

c) Representative images of tumour spheres (TS) arising from GIC19 (left) and 31 (right) cultured in non-adherent condition and treated with vehicle (top) or DSF 1µM (bottom) after 4 days of treatments. Scale bar is 20µm. Experiments have been performed on each line 3 independent times with consistent results.

d) Measurement of ALDH activity: FACS plots (top) showing ALDH FITC-labelled without and with ALDH inhibitor: N,N-diethylaminobenzaldehyde (DEAB). Quantification of ALDH activity (bottom) measured as difference between FITC + cells with and without DEAB after 4 days of treatment with vehicle (black bars) or DSF 1µM (orange hatched pattern bars) in GIC19 (left) and 31 (right). (n=2 patients, experiment was repeated 3 times, one-way ANOVA, *p* value (GIC19+DSF vs GIC 31+DSF)=0,0187).

e) Western blot analysis of ALDH3B1 upon knock-down in GIC19 (shALDh3B1) (right) as compared to control (scramble) (left), Tubulin is used as housekeeping protein. Experiments have been performed 3 independent time with consistent results.

f) mRNA expression of *ALDH* isoforms in GIC19 upon *ALDH3B1* silencing (orange bars) standardised on GAPDH expression level on expression level of GIC19 control (scramble) (black bar), n=3 independent transductions, one-way ANOVA, *p* value (*ALDH3B1*)<0,0001.

g) Proliferation assay of GIC19 scramble (black line) and sh*ALDH3B1* (orange line) with results expressed as fold change of cell number at day 10 on number of plated cells, n=3 repetitions of experiment, two tailed *t* test, *p* value=0,0226.

h) Proliferation assay of GIC19 scramble (black line) and sh*ALDH3B1* (orange line) after 4 days of treatment with DSF 1 µM (orange dots) standardised on vehicle treated cells (n=3 repetitions of experiment, two-way ANOVA, *p* value (scramble)=0,0042, *p* value (sh*ALDH3B1*)=0,7959).

All graphs report mean ±SEM. Statistical significance for all panels *$p \leq 0.05$, **$p \leq 0.01$, ***$p \leq 0.001$. Source data are provided in the source data file.

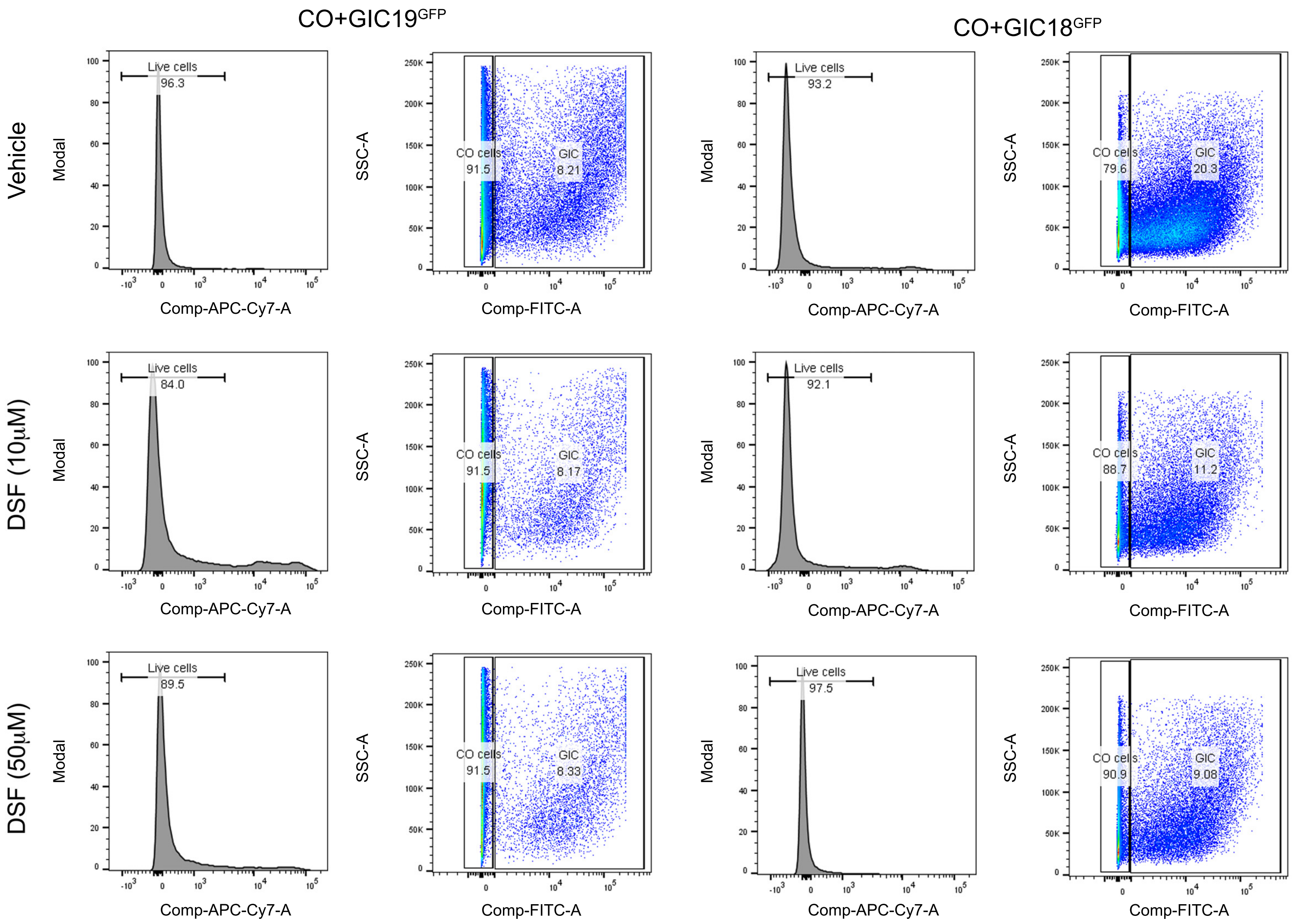
CO+GIC19GFP
CO+GIC18GFP
Vehicle
DSF (10μM)
DSF (50μM)
Modal
SSC-A
Comp-APC-Cy7-A
Comp-FITC-A
Live cells
96.3
93.2
84.0
92.1
89.5
97.5
CO cells
91.5
91.5
91.5
79.6
88.7
90.9
GIC
8.21
8.17
8.33
20.3
11.2
9.08

Fig. S13: ALDH3B1/DSF treatment characterisation in syngeneic GLICO model.

Representative FACS plots of overall viability of the organoids analysed 4 days post-treatment with vehicle or DSF (10µM, 50µM) after gating for Zombie NIR^{TM} negative cells (live cells) shown on plots as histogram of Comp-APC-Cy7-A, and total GFP population after gating for FITC, from which the GFP MFI was calculated. Cerebral Organoids (CO)+GIC19^{GFP}(left) and control patient: CO+GIC18^{GFP}(right).

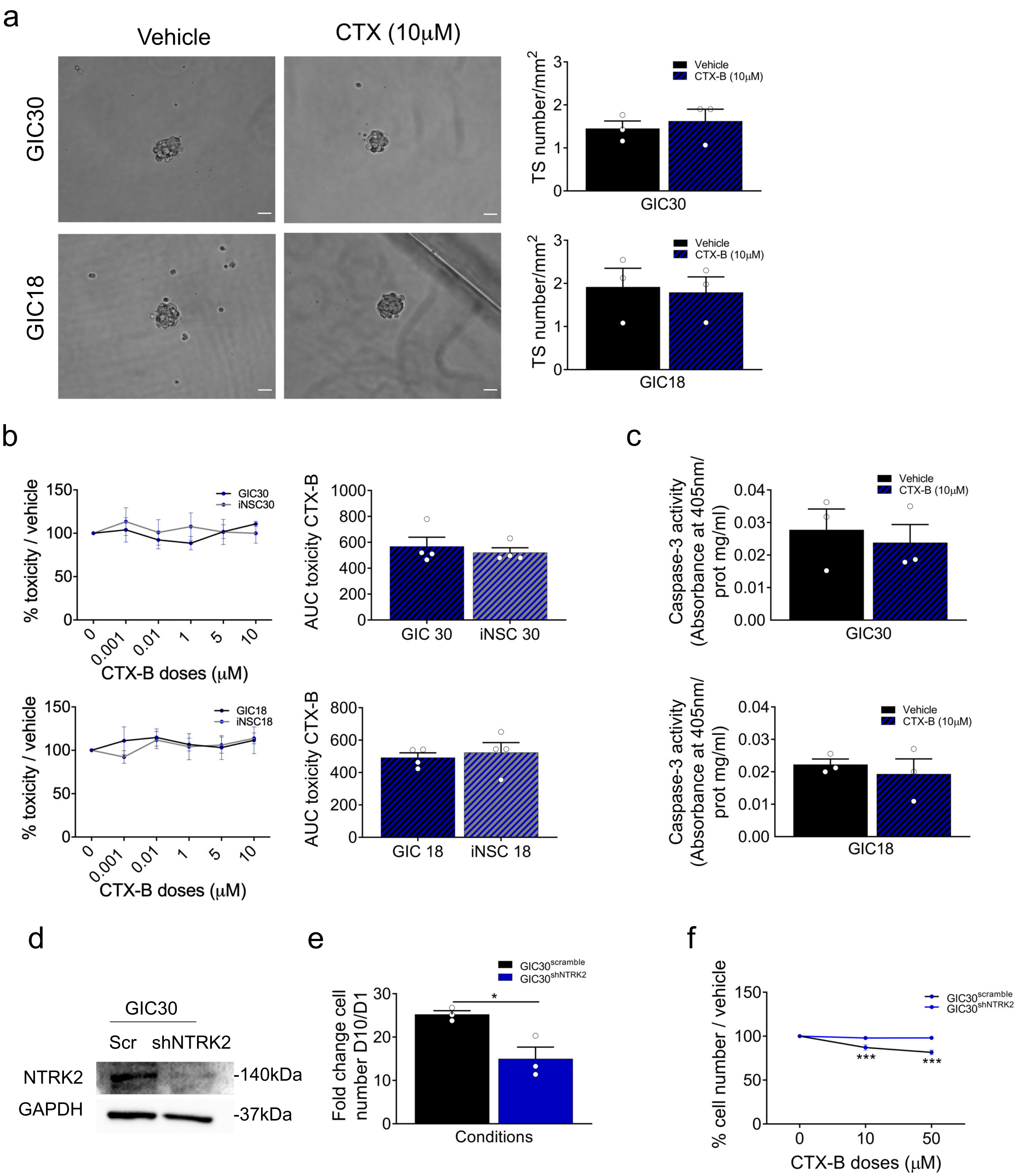

a
Vehicle
CTX (10μM)
GIC30
GIC18
TS number/mm2
Vehicle
CTX-B (10μM)
b
% toxicity / vehicle
GIC30
iNSC30
CTX-B doses (μM)
AUC toxicity CTX-B
GIC 30
iNSC 30
GIC18
iNSC18
GIC 18
iNSC 18
c
Caspase-3 activity (Absorbance at 405nm/ prot mg/ml)
d
GIC30
Scr
shNTRK2
NTRK2
GAPDH
-140kDa
-37kDa
e
GIC30scramble
GIC30shNTRK2
Fold change cell number D10/D1
Conditions
f
% cell number / vehicle
CTX-B doses (μM)

Fig. S14: NTRK2/CTX-B characterisation in 2D culture.

a) Representative images of tumour spheres (TS) arising from GIC30 (top) and 18 (bottom) cultured in non-adherent condition and treated with vehicle (left) or CTX-B 10µM (right) after 4 days of treatments (left) (scale bar is 20µm) and quantification of TS number per area (n=3 repetitions of experiment each , two tailed *t* test, *p* value (GIC30)=0,6269, *p* value (GIC18)=0,8347. Experiments have been performed 3 independent times with consistent results.

b) Drug treatment on GIC (black curves) and iNSC (grey curves) of patient 30 (left) and 18 (middle) with a range of doses from 1nM to 10µM of CTX-B (blue dots). Results are expressed in percentage of cytoxicity on the vehicle and were measured at end point, area under the curve (AUC) was calculated from percentages of toxicity (n=4 repetitions of experiment, two tailed *t* test, *p* value (GIC30)=0,5754, *p* value (GIC18)=0,6605).

c) Apoptosis as assessed by Caspase 3 activity, was measured after 4 days of treatment with vehicle (black bars) or CTX-B 10µM (blue hatched pattern) in GIC30 (top) and 18 (bottom) (n=3 repetitions of experiment, two tailed, *p* value (GIC30)=0,6675, *p* value (GIC18)=0,5849).

d) Western blot analysis of NTRK2 expression in GIC30 scramble and GIC30 upon silencing (shNTRK2). GAPDH is used as housekeeping protein.

e) Proliferation assay of GIC30 scramble (black bar) and sh*NTRK2* (blue bar) with results expressed in fold change of cell number at day 10 on cells number at D1 (n=3 repetitions of experiment, two tailed *t* test, *p* value=0,0227).

f) Proliferation assay of GIC30 scramble (black line) and sh*NTRK2* (blue line) after 4 days of treatment with CTX-B 10µM (blue dots) standardised on vehicle treated cells (n=3 repetitions of experiment,two-way ANOVA, Scramble: *p* value (10µM)<0,0001, *p* value (50µM)<0,0001; sh*NTRK2* *p* value (10µM)=0,6011, *p* value (50µM)=0,7658).

All graphs report mean ±SEM. Statistical significance for all panels **p*≤0.05, ***p*≤0.01, ****p*≤0.001. Source data are provided in the source data file.

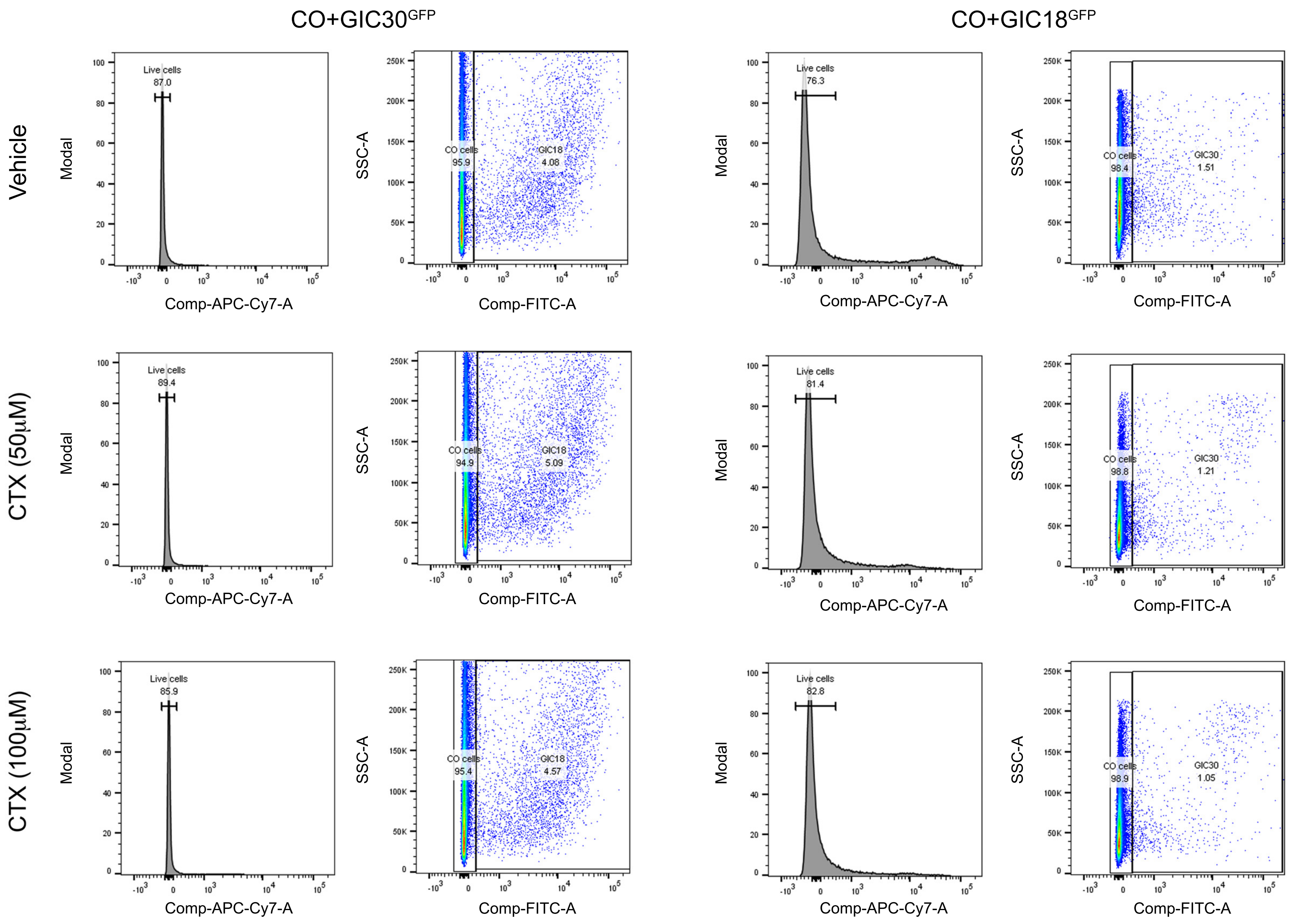
CO+GIC30GFP
CO+GIC18GFP
Vehicle
CTX (50µM)
CTX (100µM)
Live cells
87.0
89.4
85.9
76.3
81.4
82.8
CO cells
95.9
94.9
95.4
98.4
98.8
98.9
GIC18
4.08
5.09
4.57
GIC30
1.51
1.21
1.05
Modal
SSC-A
Comp-APC-Cy7-A
Comp-FITC-A

Fig.S15 ***NTRK2/CTX-B treatment characterisation in SYNGLICO***

Representative FACS plots of overall viability of the SYNGLICO (Cerebral Organoids (CO)+GIC^{GFP}) 4 days post-treatment with vehicle or CTX-B (50µM, 100µM) after gating for Zombie NIR^{TM} negative cells (live cells) shown on plots as histogram of Comp-APC-Cy7-A, and total GFP population after gating for FITC, from which the GFP MFI was calculated. CO+GIC30^{GFP} (left) and control patient: CO+GIC18^{GFP} (right).

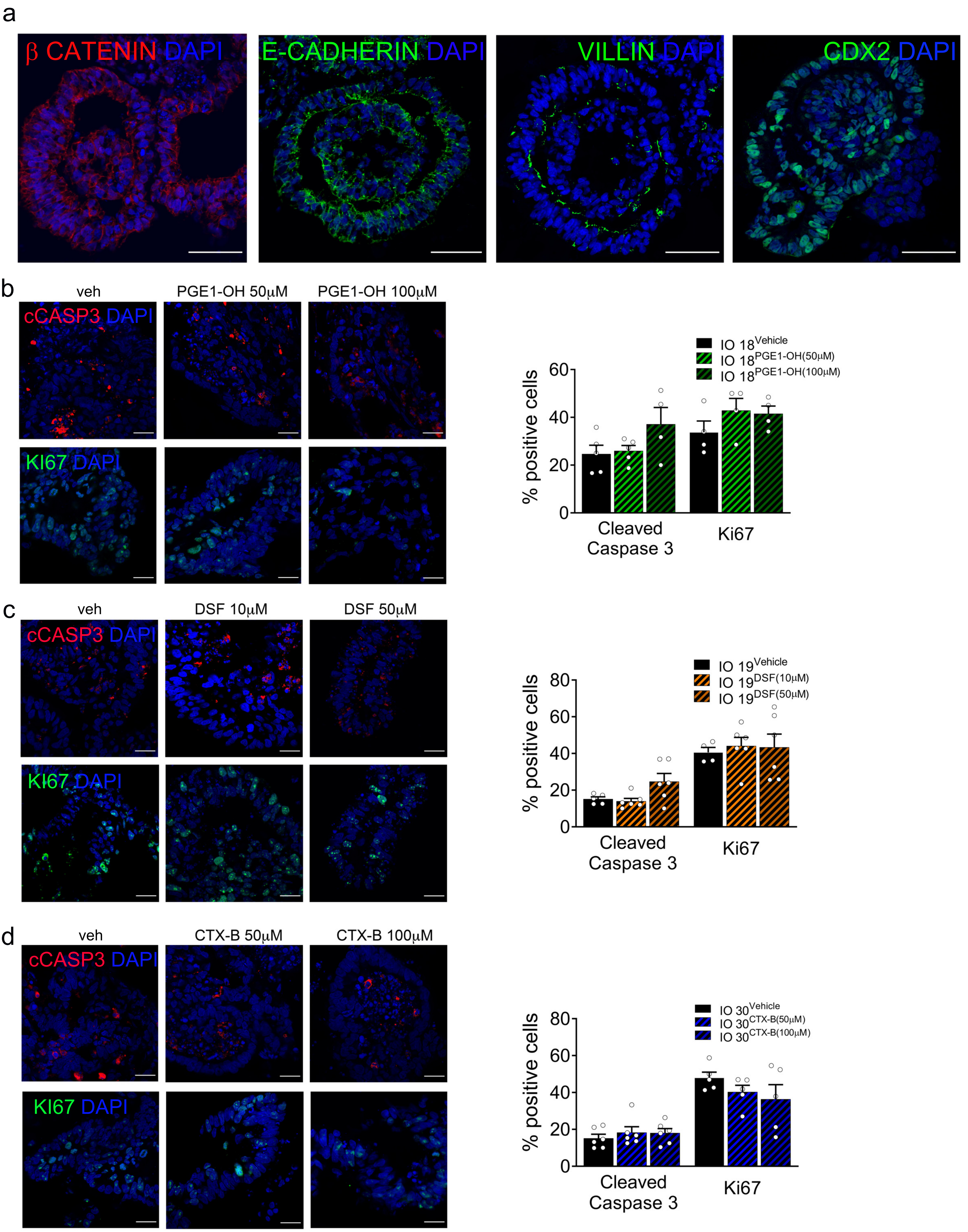
a
β CATENIN DAPI
E-CADHERIN DAPI
VILLIN DAPI
CDX2 DAPI
b
veh
PGE1-OH 50μM
PGE1-OH 100μM
cCASP3 DAPI
KI67 DAPI
IO 18^{Vehicle}
IO 18$^{PGE1-OH(50μM)}$
IO 18$^{PGE1-OH(100μM)}$
% positive cells
0
20
40
60
Cleaved Caspase 3
Ki67
c
veh
DSF 10μM
DSF 50μM
cCASP3 DAPI
KI67 DAPI
IO 19^{Vehicle}
IO 19$^{DSF(10μM)}$
IO 19$^{DSF(50μM)}$
% positive cells
0
20
40
60
80
Cleaved Caspase 3
Ki67
d
veh
CTX-B 50μM
CTX-B 100μM
cCASP3 DAPI
KI67 DAPI
IO 30^{Vehicle}
IO 30$^{CTX-B(50μM)}$
IO 30$^{CTX-B(100μM)}$
% positive cells
0
20
40
60
80
Cleaved Caspase 3
Ki67

Fig. S16 Gastro-intestinal drug toxicity assay in patient-specific EPSC derived intestinal organoids.

a) Representative images of EPSC-derived intestinal organoids (SYNIO) immunostained for β CATENIN, E-CADHERIN, VILLIN and CDX2 (from left to right), nuclei were counterstained with DAPI. Scale bar is 50µm. Experiments have been performed on 3 IO (from patient 18, 19 and 30) with consistent results. b-d) Representative images of EPSC-derived intestinal organoids (SYNIO) from patient 30 (b), 19 (c) and 30 (d) stained with cleaved caspase 3 (aCasp3) (top) or KI67 (bottom) after 4 days of treatment with vehicle or PGE1-OH (50µM and 100µM) (B), DSF (10µM and 50µM) (c) and CTX-B (50µM and 100µM) (d). Quantification results (right) expressed as percentage of KI67or cCasp3 positive cells on the total number of cells per field (PGE1-OH treatment: Ki67 vehicle and 50µM n=5, all other conditions n=4; CTX-B: vehicle cCas3 n=4, vehicle Ki67 n=5, all other conditions n=6; DSF treatment: cCas3 n=5, Ki67 n=6, one-way ANOVA, b) cCasp3: *p* value (Veh vs 100µM)=0,1606, Ki67: *p* value (Veh vs 100µM)=0,4586; c) cCasp3: *p* value (Veh vs 50µM)=0,0971 , Ki67: *p* value (Veh vs 50µM)=0,9444, d) cCasp3: *p* value (Veh vs 100µM)=0,7236 , Ki67: *p* value (Veh vs 100µM)=0,3102.

All graphs report mean ±SEM. Statistical significance for all panels *$p \leq 0.05$, **$p \leq 0.01$, ***$p \leq 0.001$. Source data are provided in the source data file.
