# Supplementary material for: Comparative epigenetic analysis of tumour initiating cells and syngeneic EPSC-derived neural stem cells in glioblastoma
Source: Nat Commun. 2021 Oct 21;12:6130. doi: 10.1038/s41467-021-26297-6 (PMC8531305; doi:10.1038/s41467-021-26297-6)
Supplement: Supplementary file 3 — Description of Additional Supplementary Files [file 41467_2021_26297_MOESM3_ESM.docx]

Description of Additional Supplementary Files

Title: Supplementary Data 1

Description: Tabular results for combined DE and DMR analyses covering all genes represented in both modalities (11858 in total). Each gene (identified by Ensembl gene ID and gene symbol) is associated with one or more DMRs (identified by an arbitrary DMR cluster number). The columns 1stExon through to TSS200 indicate how the DMR is associated with the gene based on its genomic locus as follows: within the 1st exon, in the 3' or 5' untranslated region (UTR), in the gene body, at the exon boundary, within 1500 nucleotides of the transcription start site (TSS) and within 200 nucleotides of the TSS. One or more of these is true for each (DE, DMR) pair.

The extent of differential expression between GIC and iNSC in each patient is given in the columns <patient_id>_de_logfc, quantified as the log2 fold change. Red shading indicates an increase of expression in GIC, green shading indicates a decrease. Black borders indicate comparisons for which the adjusted *p* value is below 0.05.

The extent of differential methylation within DMRs is given in the columns <patient_id>_dm_delta, quantified as the median difference in *M* value between GIC and iNSC across all probes in the DMR. Red shading indicates hypermethylation in GIC, green indicates hypomethylation. Black borders indicate comparisons for which the adjusted *p* value is below 0.05.

Within the DE and DMR blocks, bold text indicates concordance between DE and DMR values, defined as differing signs. Italic text indicates discordance (equal signs).

The num_DGIdb_interactions column displays how many known drug compounds are listed against this gene in the drug gene interaction database. Comments list the compounds; more information is available in the DGIdb worksheet. Finally, the list_member column indicates which list, if any, a given (DE, DMR) pair belongs to. Further details of the short and long lists are given in the results section of the manuscript.
