# Supplementary material for: Comparative epigenetic analysis of tumour initiating cells and syngeneic EPSC-derived neural stem cells in glioblastoma
Source: Nat Commun. 2021 Oct 21;12:6130. doi: 10.1038/s41467-021-26297-6 (PMC8531305; doi:10.1038/s41467-021-26297-6)
Supplement: Supplementary file 5 — Reporting Summary [file 41467_2021_26297_MOESM5_ESM.pdf]

nature research

Corresponding author(s): Silvia Marino

Last updated by author(s): Jul 23, 2021

Reporting Summary

Nature Research wishes to improve the reproducibility of the work that we publish. This form provides structure for consistency and transparency in reporting. For further information on Nature Research policies, see our Editorial Policies and the Editorial Policy Checklist.

Statistics

For all statistical analyses, confirm that the following items are present in the figure legend, table legend, main text, or Methods section.

n/a	Confirmed	
☐	☒	The exact sample size (*n*) for each experimental group/condition, given as a discrete number and unit of measurement
☐	☒	A statement on whether measurements were taken from distinct samples or whether the same sample was measured repeatedly
☐	☒	The statistical test(s) used AND whether they are one- or two-sided<br>*Only common tests should be described solely by name; describe more complex techniques in the Methods section.*
☐	☒	A description of all covariates tested
☐	☒	A description of any assumptions or corrections, such as tests of normality and adjustment for multiple comparisons
☐	☒	A full description of the statistical parameters including central tendency (e.g. means) or other basic estimates (e.g. regression coefficient) AND variation (e.g. standard deviation) or associated estimates of uncertainty (e.g. confidence intervals)
☐	☒	For null hypothesis testing, the test statistic (e.g. *F*, *t*, *r*) with confidence intervals, effect sizes, degrees of freedom and *P* value noted<br>*Give P values as exact values whenever suitable.*
☒	☐	For Bayesian analysis, information on the choice of priors and Markov chain Monte Carlo settings
☐	☒	For hierarchical and complex designs, identification of the appropriate level for tests and full reporting of outcomes
☐	☒	Estimates of effect sizes (e.g. Cohen's *d*, Pearson's *r*), indicating how they were calculated

Our web collection on statistics for biologists contains articles on many of the points above.

Software and code

Policy information about availability of computer code

Data collection	Details in "Material and Methods" in relevant sections
Data analysis	Details in "Material and Methods" in relevant sections

For manuscripts utilizing custom algorithms or software that are central to the research but not yet described in published literature, software must be made available to editors and reviewers. We strongly encourage code deposition in a community repository (e.g. GitHub). See the Nature Research guidelines for submitting code & software for further information.

Data

Policy information about availability of data

All manuscripts must include a data availability statement. This statement should provide the following information, where applicable:

- Accession codes, unique identifiers, or web links for publicly available datasets
- A list of figures that have associated raw data
- A description of any restrictions on data availability

The authors declare that all the data supporting the findings of this study are available within the Article and its Supplementary Information files or from the corresponding author upon reasonable request.

Field-specific reporting

Life sciences study design

All studies must disclose on these points even when the disclosure is negative.

Sample size	Details in "Material and Methods" in relevant sections
Data exclusions	No data were excluded
Replication	Experiments were repeated with biological replica (n). Number of n specified for each experiments in Figure Legend.
Randomization	Details in "Material and Methods" in relevant sections
Blinding	Data collection and analysis were conducted blinded where applicable. Non blinded analysis were conducted for in vitro and in vivo treatments requiring multiple round of administrations.

Reporting for specific materials, systems and methods

We require information from authors about some types of materials, experimental systems and methods used in many studies. Here, indicate whether each material, system or method listed is relevant to your study. If you are not sure if a list item applies to your research, read the appropriate section before selecting a response.

Materials & experimental systems

n/a	Involved in the study	
☐	☒	Antibodies
☐	☒	Eukaryotic cell lines
☒	☐	Palaeontology and archaeology
☐	☒	Animals and other organisms
☐	☒	Human research participants
☒	☐	Clinical data
☒	☐	Dual use research of concern

Methods

n/a	Involved in the study	
☒	☐	ChIP-seq
☐	☒	Flow cytometry
☒	☐	MRI-based neuroimaging

Antibodies

Antibodies used	Details in "Material and Methods" in the relevant sections
Validation	Antibodies have been commercially validated

Eukaryotic cell lines

Policy information about cell lines

Cell line source(s)	Details in "Material and Methods" in "human cell cultures" and "Animal procedures and murine cell cultures" sections
Authentication	Cells used were commercially available or authenticated when derived in the context of this study
Mycoplasma contamination	Cells tested negative
Commonly misidentified lines (See ICLAC register)	N/A

Animals and other organisms

Policy information about studies involving animals; ARRIVE guidelines recommended for reporting animal research

Laboratory animals	Details in "Material and Methods" in "Animal procedures and murine cell cultures" sections
Wild animals	N/A
Field-collected samples	N/A
Ethics oversight	Details in "Material and Methods" in "Animal procedures and murine cell cultures" sections

Note that full information on the approval of the study protocol must also be provided in the manuscript.

Human research participants

Policy information about studies involving human research participants

Population characteristics	Details in "Table S1".
Recruitment	Details in "Material and Methods" in "human cell cultures" section and "Table S1"
Ethics oversight	Details in "Material and Methods" in "human cell cultures" section and "Table S1"

Note that full information on the approval of the study protocol must also be provided in the manuscript.

Flow Cytometry

Plots

Confirm that:

- [x] The axis labels state the marker and fluorochrome used (e.g. CD4-FITC).
- [x] The axis scales are clearly visible. Include numbers along axes only for bottom left plot of group (a 'group' is an analysis of identical markers).
- [x] All plots are contour plots with outliers or pseudocolor plots.
- [x] A numerical value for number of cells or percentage (with statistics) is provided.

Methodology

Sample preparation	Details in "Material and Methods" in "Flow cytometry" section
Instrument	Details in "Material and Methods" in "Flow cytometry" section
Software	Details in "Material and Methods" in "Flow cytometry" section
Cell population abundance	Details in "Material and Methods" in "Flow cytometry" section
Gating strategy	Details in "Material and Methods" in "Flow cytometry" section

- [x] Tick this box to confirm that a figure exemplifying the gating strategy is provided in the Supplementary Information.
